# Single-cell profiling reveals distinct populations of tumor-associated macrophages and metastatic tumor cells in breast cancer brain metastasis

**DOI:** 10.1038/s41419-026-08807-w

**Published:** 2026-04-25

**Authors:** Haoyuan Shi, Jiaxin Chen, Zisheng Wu, Dong-Xu Liu, Jinmei Zhou, Xuexue Wu, Ailing Yang, Shanhu Li, Yanhong Tai, Zefei Jiang, Zhiyuan Hu, Mingshan Zhang, Xiaojie Xu, Lu Pan, Tao Wang

**Affiliations:** 1 Department of Urology, National Cancer Center/National Clinical Research Center for Cancer/Cancer Hospital, Chinese Academy of Medical Sciences and Peking Union Medical College, Beijing, China; 2 National Key Laboratory of Advanced Biotechnology, Academy of Military Medical Sciences, Beijing, China; 3 Medical School of Chinese PLA, Beijing, China; 4 Senior Department of Oncology, Chinese PLA General Hospital, Beijing, China; 5 Department of Breast Surgery; Shandong Key Laboratory of Cancer Digital Medicine; Institute of Translational Medicine of Breast Disease Prevention and Treatment; and Shandong Provincial Engineering Laboratory of Translational Research on Prevention and Treatment of Breast Disease, The Second Hospital of Shandong University, Jinan, China; 6 Pathology Department, The Fifth Medical Center of PLA General Hospital, Beijing, China; 7 CAS Key Laboratory for Biomedical Effects of Nanomaterials and Nanosafety, CAS Key Laboratory of Standardization and Measurement for Nanotechnology, CAS Center for Excellence in Nanoscience, National Center for Nanoscience and Technology, Beijing, China; 8 School of Future Medicine, Beijing University of Chinese Medicine, Beijing, China; 9 School of Nanoscience and Technology, Sino-Danish College, University of Chinese Academy of Sciences, Beijing, China; 10 Fujian Provincial Key Laboratory of Brain Aging and Neurodegenerative Diseases, School of Basic Medical Sciences, Fujian Medical University, Fuzhou, China; 11 School of Chemical Engineering and Pharmacy, Wuhan Institute of Technology, Wuhan, China; 12 Department of Neurosurgery, Sanbo Brain Hospital, Capital Medical University, Beijing, China; 13 The Second of Clinical Medicine, Southern Medical University, Guangzhou, China; 14 Anhui Medical University, Hefei, China

**Keywords:** Breast cancer, Tumour immunology, Biomarkers

## Abstract

Breast cancer brain metastasis (BCBM) is a major cause of breast cancer-related mortality, but the molecular mechanisms underlying its progression remain poorly understood. Here, we profiled the tumor immune microenvironment of BCBM at single-cell resolution and identified candidate regulators associated with brain metastatic progression. Single-cell RNA sequencing (scRNA-seq) was performed on brain metastatic tissue, adjacent tumor tissue, cerebrospinal fluid, and circulating tumor cells (CTCs) from seven patients, and bulk DNA sequencing was conducted on primary breast tumors, peripheral blood, and brain metastases from 47 patients. Analysis of 131,880 single cells identified 63 distinct cell clusters, including 12 tumor-associated macrophage (TAM) subtypes and 5 metastatic tumor cell (MTC) subtypes. Circulating TAMs displayed an M1-like inflammatory phenotype, whereas tissue-resident TAMs were predominantly M2-like. MTCs exhibited substantial transcriptional heterogeneity, and a neuro-related subtype showed adaptive upregulation of neuronal signaling pathways. Brain metastases also harbored a higher mutational burden than primary tumors, with recurrent mutations in GABRB3 and NRXN1 associated with poorer patient survival. Functional experiments further showed that loss of GABRB3 or NRXN1 impaired tumor growth and brain colonization in xenograft models. These findings nominate GABRB3 and NRXN1 as candidate regulators of brain metastatic fitness in BCBM and support their further evaluation as biomarkers and therapeutic targets.

## Introduction

Breast cancer brain metastasis (BCBM) is a relatively common clinical event [1, 2], closely linked to the molecular phenotype of the tumor [3, 4]. Patients with central nervous system (CNS) metastases face significantly reduced overall survival (OS) compared to those without such metastases [5, 6]. Despite this, routine brain imaging for the detection of brain metastasis is not a standard practice [7–9], suggesting that the actual incidence of brain metastasis may be higher than currently reported.

Recent studies indicate that approximately 50% of breast cancer patients with metastasis are HER2-positive [10–12]. Although brain metastases and primary tumors share a common ancestry, continuous tumor cell proliferation leads to numerous clonal subpopulations, increasing genetic divergence between them [13–15]. Notably, while genetic heterogeneity exists between primary tumors and brain metastases, there is a high degree of genetic homogeneity among brain metastases at different sites [13]. This suggests that brain metastatic cells share remarkably similar genomic profiles, highlighting that therapeutic strategies for BCBM cannot rely solely on the molecular subtyping of the primary tumors. Despite advances in single-cell RNA sequencing (scRNA-seq) that have significantly expanded our understanding of the brain metastatic niche across various cancer types [16], our knowledge of BCBM remains limited. It is widely accepted that brain metastases form through the seeding of circulating tumor cells (CTCs) to the brain’s microvasculature [10]. However, the CNS is protected by barriers such as the blood-brain barrier (BBB) and the blood cerebrospinal fluid barrier (BFB), which defend against the entry of foreign substances, making CTC homing in the brain a complex process [10]. A deeper understanding of the genetic, transcriptional, and tumor immune microenvironment (TIME) involved in the targeted colonization of the brain by CTCs could contribute to early detection of BCBM and the development of novel therapeutic approaches to targeting brain metastases in breast cancer patients.

In this study, we collected surgical specimens from critical sites along the primary routes of BCBM and performed scRNA-seq to investigate the TIME associated with brain metastasis. By integrating bulk DNA sequencing and The Cancer Genome Atlas Program (TCGA) datasets, we identified key mutated genes in metastatic tumor cells (MTCs) associated with brain metastasis and examined the role of GABRB3 and NRXN1 in modulating BCBM progression.

## Subjects and methods

### Patient sample collection

DNA sequencing data were collected from various specimens, including breast cancer primary tissues, peripheral whole blood (before the surgery), and brain metastatic tumor tissues. These specimens were surgically resected at the Fifth Medical Center of the PLA General Hospital between 2004 and 2020. The brain metastatic tumor tissues included paraffin-embedded samples and fresh surgically resected samples; the breast cancer primary tissues were paraffin specimens. Additionally, single-cell transcriptome sequencing data of brain metastatic tumor tissues and CTCs were obtained from fresh surgical samples collected between March 2023 and August 2023. For further details, see the Supplementary Methods.

### Library construction and sequencing

Library construction and single-cell sequencing were performed as detailed in Supplementary Methods.

### Cell annotation

To identify major cell types, we used the Seurat R package [17–21]. To distinguish brain-resident microglia from monocyte-derived macrophages, we applied established lineage-specific markers. Microglia were identified by the expression of TMEM119, P2RY12, and SALL1, together with homeostatic markers such as CX3CR1 and CSF1R, whereas peripherally derived macrophages were defined by markers including LYZ, CD163, AIF1, LST1, SPP1, and APOE. A full marker list used for cell-type annotation is provided in the Supplementary Methods. For details, see the Supplementary Methods.

### Copy number variation analysis

Copy number variation (CNV) analysis was conducted on scRNA-seq data using the inferCNV R package [22] as detailed in Supplementary Methods.

### Pathway enrichment analyses

Gene Ontology (GO) Enrichment Analysis: To identify enriched GO biological processes among DEGs for each cell type, we utilized the clusterProfiler::gseGO function from the clusterProfiler R package [23] with gene annotations from org.Hs.eg.db. See more details in Supplementary Methods.

### Single-cell level somatic mutations calling

Somatic mutation analysis at the single-cell level was conducted using the Numbat R package [24]. For somatic mutation analysis at the cell type level, we followed the protocol of SComatic [25]. For details, see the Supplementary Methods.

### Developmental trajectory and RNA velocity analysis

Monocle3 [26–28] and Velocyto [29] were adopted to calculate and infer the developmental trajectory and RNA velocity of MTCs and tumor-associated macrophages (TAMs). Spliced and unspliced UMIs for each gene in each cell were quantified using the Velocyto Python package, and downstream analyses were performed with scvelo [30]. See Supplementary Methods for further details

### Cell–cell communication analysis

Cell–cell communication analysis was conducted on the scRNA-seq data using the CellChat [31] software as detailed in Supplementary Methods.

### TCGA survival analysis

Survival analysis was conducted using the UCSC Xena platform [32] with gene expression profiles from the TCGA-BRCA dataset in HTSeq-FPKM format. Kaplan–Meier survival curves were plotted using the ggsurvplot function from the Survminer R package. For TCGA-BRCA survival analyses, patients were stratified by the mean mRNA expression level of each gene, and associations with OS were examined using Kaplan–Meier curves and two-sided log-rank tests, with hazard ratios estimated from univariable Cox proportional hazards models.

For details of in vitro and in vivo experiments, see Supplementary Methods.

### Ethics

This study was reviewed and approved by the Ethics Committee of the Fifth Medical Center of the Chinese People’s Liberation Army General Hospital (ethics approval number: KY-2021-7-48-1). It was conducted in accordance with the principles of the Declaration of Helsinki and the International Conference on Harmonization Guidelines for Good Clinical Practice. Written informed consent was obtained from all patients whose human specimens were collected. All animal experiments were approved and guided by the Ethics Committee of the Beijing Institute of Biotechnology.

## Results

### scRNA-seq reveals distinct metastatic tumor cell types, microglia, and tumor-associated macrophages in breast cancer brain metastasis

**Fig. 1:**
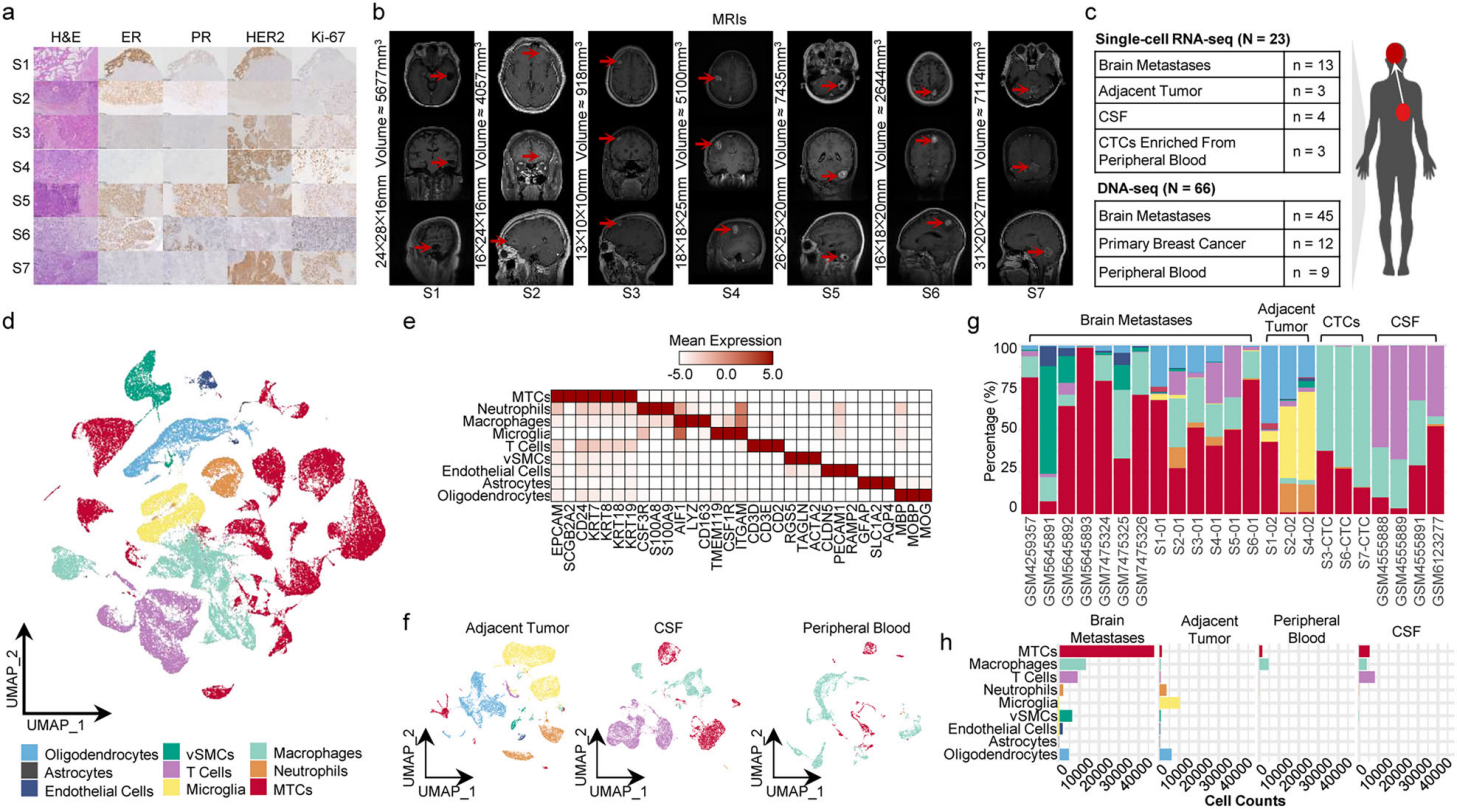
Identification of various cell types in brain metastases from breast cancer. **a** Representative images of hematoxylin and eosin (H&E) staining and immunohistochemical (IHC) analysis of key breast cancer biomarkers in tumor samples from seven patients (S1–S7). **b** Representative magnetic resonance imaging (MRI) of brain metastases with tumor sites indicated by red arrows. **c** Sample sizes for single-cell RNA sequencing (scRNA-seq) and DNA sequencing (DNA-seq) from each sampling site. **d** Uniform manifold approximation and projection (UMAP) plots showing nine major cell types, with individual dots representing cells. Colors correspond to distinct cell clusters. **e** Relative mean expression levels of key gene markers across the major cell types. **f** UMAP plots showing the distribution of major cell types, grouped by sampling site. **g** Stacked bar plots depicting the distribution of major cell types across each sample. **h** Bar plots representing the count of each cell type at various sampling sites. CTC circulating tumor cells, CSF cerebrospinal fluid, ER estrogen receptor, PR progesterone receptor, HER2 human epidermal growth factor receptor 2, vSMCs vascular smooth muscle cells, MTCs metastatic tumor cells.

To investigate brain metastasis in breast cancer, we recruited seven patients with breast cancer (six female and one male) for the study (Supplementary Tables 1 and 2). Tumor morphology was examined through hematoxylin and eosin (H&E) staining (Fig. 1a), and the expression of key breast cancer biomarkers—estrogen receptor (ER), progesterone receptor (PR), HER2, and Ki-67—was assessed by immunohistochemical (IHC) analysis (Fig. 1b). Brain metastasis was confirmed by MRI (Fig. 1c).

We conducted scRNA-seq on brain metastatic tissues (*n* = 6), adjacent tumor tissue (*n* = 3), and enriched CTCs of peripheral blood (*n* = 3) collected from these seven patients. To enhance the dataset’s robustness, we included scRNA-seq data from previous studies on brain metastatic tissues by Gonzalez et al. (*n* = 3) [16], Song et al. (*n* = 3) [33], Wang et al. (*n* = 1) [34], and cerebrospinal fluid (CSF) by Chi et al. (*n* = 3) [35] and Ruan et al. (*n* = 1) [36], bringing the total number of scRNA-seq samples to 23 (Fig. 1d, upper panel).

Additionally, we performed bulk DNA-seq of paraffin-embedded tissues from 47 BCBM patients (Supplementary Tables 3 and 4). DNA-seq data were obtained from primary tumors (*n* = 12), peripheral blood (*n* = 9), and brain metastases (*n* = 45) (Fig. 1d, lower panel). After rigorous quality control (Supplementary Fig. 1), RNA-seq analysis identified 131,880 single cells. The scRNA-seq data were analyzed using a custom computational pipeline built on the Seurat V4 R package [19]. Unsupervised clustering revealed 63 distinct cell clusters, which were classified into nine major cell types: MTCs, macrophages, T cells, oligodendrocytes, microglia, vascular smooth muscle cells (vSMCs), neutrophils, endothelial cells, and astrocytes (Fig. 1d). These cell types exhibited distinct gene expression profiles (Fig. 1e, Supplementary Fig. 3, Supplementary Table 5). Of particular interest, MTCs expressed highly specific markers—EPCAM, SCGB2A2, CD24, KRT7, KRT8, KRT18, and KRT19—that were almost entirely absent from normal brain tissue (Fig. 1e). MTCs exhibited a widely dispersed distribution across the UMAP dimensions (Supplementary Fig. 4a), highlighting significant inter-patient transcriptional heterogeneity (Supplementary Fig. 4b). Notably, MTCs from individual patients formed independent clusters, with no overlap across patients, while non-MTCs formed well-defined clusters shared across patient samples.

Furthermore, the clustering of cell types was consistent regardless of sampling site, data source, gender, or molecular phenotype (Fig. 1f, Supplementary Fig. 4c–g). The distribution of cell types varied across different sampling locations: brain metastasis samples were enriched in MTCs, adjacent tumor tissues showed a predominance of microglial cells, and blood and CSF samples contained a higher abundance of immune cells (Fig. 1g, h). Due to the negative enrichment process applied to CTCs, very few T cells were present in the blood samples, contrasting with CSF samples (Fig. 1h). Overall, the scRNA-seq data revealed that MTCs were the predominant cell type, followed by TAMs (Supplementary Fig. 5). Consistent with previously reported immune landscapes of CNS metastases, B cells and classical dendritic cells were extremely rare or undetectable in our dataset, reflecting their well-known biological scarcity in the CNS metastatic microenvironment.

### Molecular characterization of macrophages in the TIME of breast cancer brain metastasis

**Fig. 2:**
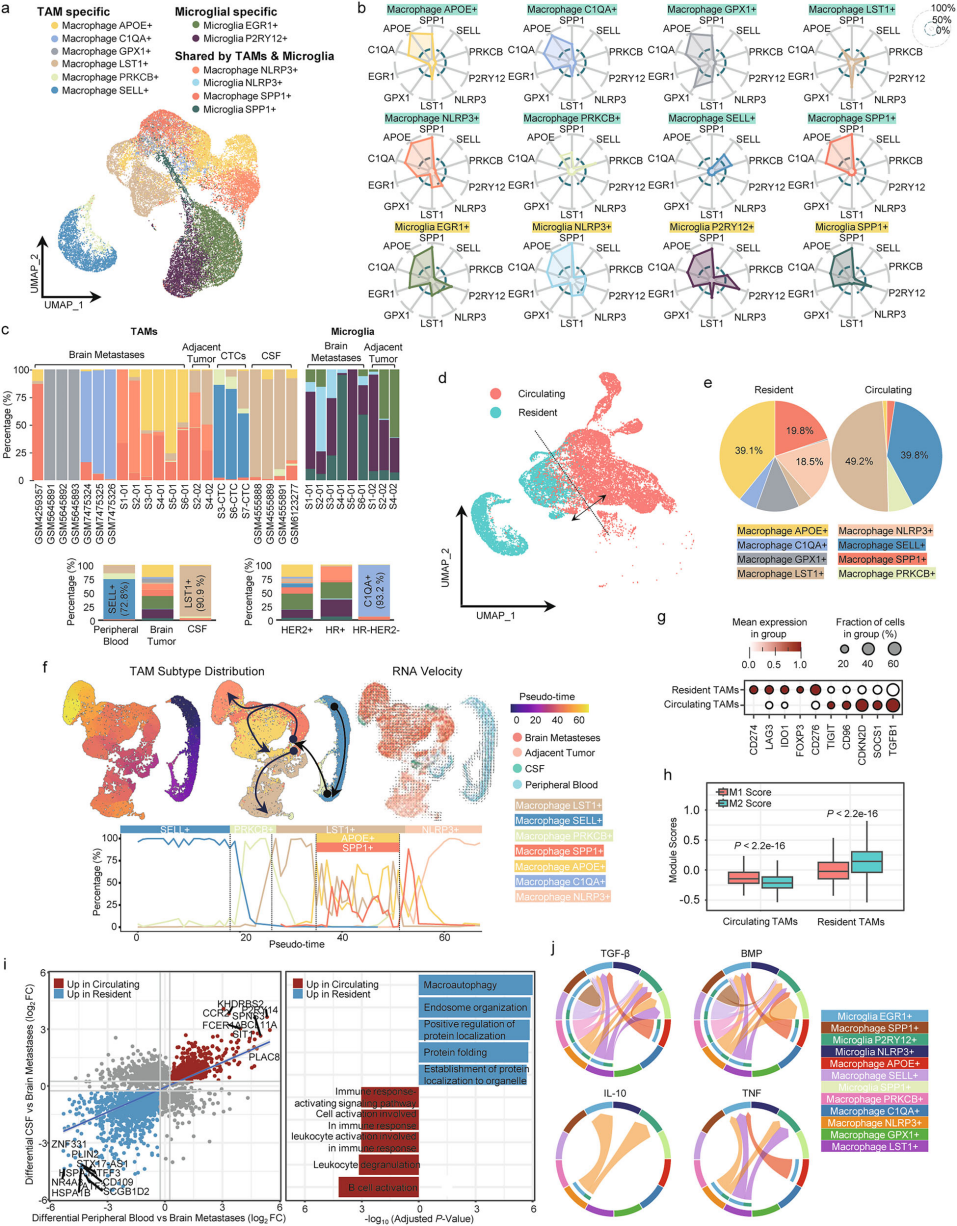
High heterogeneity of TAMs predicts a clear trajectory of their development during brain metastasis. **a** UMAP plots showing the distribution of 12 macrophage subtypes across all samples. **b** Radar plots displaying the relative mean expression of the marker genes for all the 12 macrophage subtypes, with the distance from the center indicating normalized gene expression levels (scaled from 0% to 100%). **c** Stacked bar plot showing the distribution of macrophage subtypes across different samples, sampling sites, and histopathological molecular classifications of brain metastasis samples. **d** UMAP plots showing the boundary between resident and circulating macrophages, with dashed lines indicating the classification threshold and arrows pointing to the domains of each cell type. **e** Pie plots showing the proportions of major TAM subtypes within circulating and resident populations. **f** *Top row*: pseudo-time trajectory analysis of TAM development during brain metastasis, inferred by Monocle3. Developmental paths are indicated by arrows, and RNA velocity streamlines from scVelo reflect relative RNA states along the trajectories. *Bottom row*: molecular changes observed in TAMs along the pseudo-time axis. **g** Comparison of the relative mean expression of specific gene markers between resident and circulating TAMs. **h** Box plots showing the distribution of M1/M2 module scores in TAMs between resident and circulating TAMs. **i** Differentially expressed genes (DEGs) (*left panel*) and Gene Set Enrichment Analysis (GSEA) results (*right panel*) comparing circulating TAMs from different sampling sites to resident TAMs. **j** Chord diagram illustrating the interactions between each TAM subtype and microglia, with line width representing interaction strength and color-coded by cell subtypes. The diagram is annotated with the names of intercellular signaling pathways involved. HR+ hormone receptor positive, HER2+ human epidermal growth factor receptor 2 positive, HR-HER2− HR and HER2 both negative, FC fold change.

We next investigated the TIME for brain metastasis. TIME has been shown to play a crucial role in modulating and enhancing the invasive and colonizing capabilities of MTCs [37–40]. Microglia, the resident macrophages of the CNS, serve as the first line of defense against invading pathogens and cancer cells, was reported to participate in the earliest steps of BCBM [41]. We thus performed a high-resolution subtype annotation on the 33,246 microglia and TAMs. Clustering analysis grouped macrophages into 12 distinct subtypes. These 12 clusters consisted of four brain-resident microglial subtypes and eight monocyte-derived TAM subtypes, as determined by canonical markers (microglia: TMEM119, P2RY12, SALL1; TAMs: LST1, APOE, SPP1, NLRP3, CD163) (Fig. 2a). Each of these 12 subtypes was characterized by a unique expression profile of these biomarkers (Fig. 2b). UMAP analysis demonstrated that the inter- cellular heterogeneity of externally derived TAMs from brain metastases, adjacent CTCs and CSF was significantly higher than that of brain-resident microglia (Supplementary Fig. 6), highlighting the complexity of TAMs in the context of brain metastasis. We observed substantial differences in the distribution of macrophage subtypes among patients, sample sources, and molecular subtypes of brain metastasis (Fig. 2c).

TAMs are not native to the brain but are a major component of TIME. To investigate their role, we categorized TAMs as resident and circulating subtypes based on the sample’s intracranial origin. UMAP plots revealed a clear distinction between these two groups (Fig. 2d). The most abundant TAM subtypes within the TIME were APOE (39.1%), SPP1 (19.8%), and NLRP3 (18.5%) macrophages, whereas the dominant TAM subtypes in the CSF or peripheral blood were LST1 (49.2%) and SELL (39.8%) macrophages (Fig. 2e).

To explore their developmental trajectory, we performed pseudo-time analysis on TAMs derived from brain metastasis and adjacent tumor tissues, as well as peripheral blood and CSF. This analysis identified five distinct evolutionary trajectories of TAM development during brain metastases (Fig. 2f). Along the pseudo-time axis, we observed a clear progression of TAM phases: SELL TAMs predominated at the early stage, PRKCB TAMs were more prevalent in the middle stage, and LST1, APOE, and SPP1 TAMs became more abundant in the later stages. Finally, NLRP3 TAMs emerged at the terminal stage, marking a clear diversification in the evolution of TAMs (Fig. 2f).

### Signaling signatures of TAMs during brain metastasis

We next examined the gene signatures of resident and circulating TAM subtypes. DEG analysis revealed distinct gene expression profiles between these two groups (Supplementary Table 6). Resident TAMs exhibited high expression of immunosuppressive genes, including *CD274*, *CD276*, *LAG3*, *IDO1*, and *FOXP3*, while circulating TAMs showed elevated expression of genes that negatively regulate immune responses, such as *TIGIT*, *CD96*, *CDKN2D*, *SOCS1*, and *TGFB1*. This suggests that the expression of immune response modifiers differs between resident and circulating TAM (Fig. 2g). TAMs typically express both M1-like and M2-like gene signatures, yet differences in gene expression patterns result in the manifestation of disparate biological functions. We demonstrated that circulating TAMs expressed higher levels of M1-like signatures, whereas resident TAMs displayed higher levels of M2-like signatures (Fig. 2h). This suggests that resident TAMs acquire M2-like properties to promote tissue infiltration of MTCs.

Circulating TAMs comprise two subtypes with distinct transcriptomic profiles that share differentially expressed genes with resident TAMs. These differences likely enable TAM adaptation to the brain microenvironment and support metastatic colonization. We performed differential gene expression analysis comparing peripheral blood and CSF TAMs with resident brain TAMs (Supplementary Table 7). Distinct gene signatures were identified between TAMs from CSF or peripheral blood and those from brain metastasis samples. Several genes were co-upregulated and co-downregulated in circulating TAMs (Fig. 2i, left). GSEA shows chemokine signaling dominates circulating TAMs, while transcription and synthesis pathways dominate resident TAMs (Fig. 2i, right). Circulating TAMs are migratory; resident TAMs focus on protein synthesis in the brain. CellChat reveals TAM subpopulations communicate with distinct microglial subtypes (Fig. 2j), with TAMs as ligand sources and microglia as receptors. TAMs secrete immunosuppressive TGF-β and IL-10, promoting microglial M2 polarization and suppressing anti-tumor immunity. BMP and TNF pathways regulate microglial inflammation, creating a tumor-favorable environment. This TAM-microglia crosstalk is critical for brain metastasis and represents therapeutic targets to enhance anti-tumor immunity.

### Molecular characterization of MTCs during breast cancer brain metastasis at single-cell resolution

**Fig. 3:**
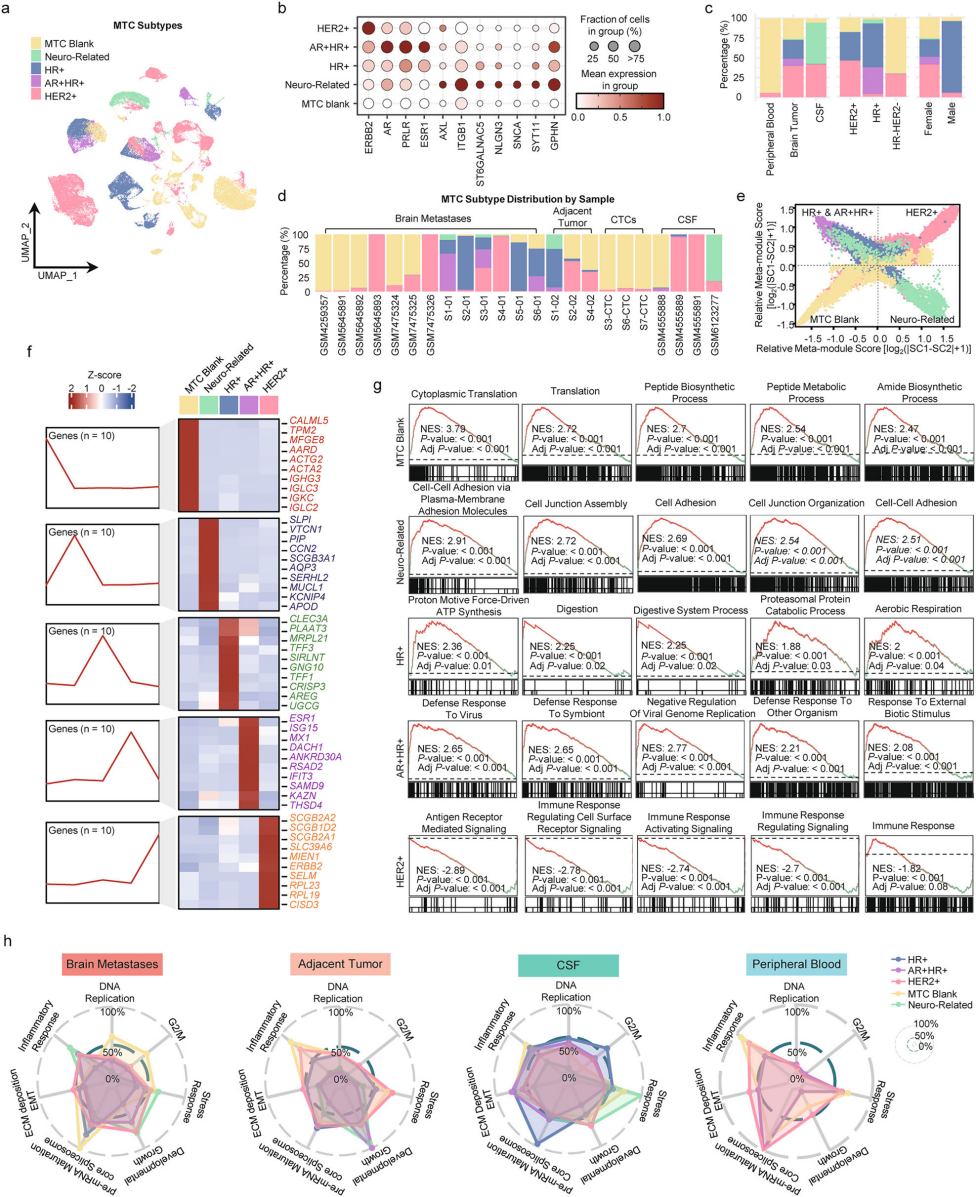
MTCs heterogeneity and biological function in each subtype. **a** UMAP plots showing the distribution of subtypes of all samples MTCs colored by cluster. **b** The relative mean expression of specific gene markers in each of the five subtypes of MTCs. Stacked bar plots showing the distribution of MTC subtypes in sampling sites, histopathological molecular classification, gender (**c**), and samples (**d**). **e** Two-dimensional butterfly plot visualization of the indicated four MTC molecular subtypes in different clusters, representing signature scores as relative meta-module scores. Each quadrant corresponds to one subtype; the exact position of each cell reflects its relative signature scores in all four subtypes. **f** Heatmap plot showing the top-10 markers of each of the MTCs subtypes. **g** Gene set enrichment analysis (GSEA) for the five subtypes of MTCs. **h** Radar plots show cancer-related pathways enriched for the five subtypes of MTCs. MTCs subtypes are denoted by colors. The distance from the dots to the center of the circle represents the normalized expression of each pathway scaled to 0–100%. NES normalized enrichment score, ECM extracellular matrix, EMT epithelial–mesenchymal transition.

MTCs possess intrinsic properties that allow them to invade and colonize distant organs, including the brain. Understanding these subtypes at the single-cell level is essential for developing effective, targeted therapies [10, 11]. We classified the MTCs into five molecular subtypes—MTC blank, neuro-related, AR+HR+, HR+, and HER2+—based on curated marker-gene signatures and meta-module scoring (Fig. 3a, b). This signature-based approach captures conserved functional programs independently of the global transcriptional background. Although MTCs exhibited pronounced inter-patient heterogeneity and formed largely patient-specific clusters in the UMAP embedding, several functional subtypes—particularly the neuro-related, HER2+, AR+HR+, and HR+ subtypes—were recurrently observed across individuals.

Among these subtypes, MTC blank and HER2+ subtypes displayed the highest inter-cell transcriptional heterogeneity, while the neuro-related subtype exhibited the least variability (Supplementary Fig. 9). Substantial differences in subtype distribution were observed across sampling sites, histopathological classifications, and patient gender (Fig. 3c, d). Tracing the trajectory of MTCs from blood to brain to CSF, we noted a decline in MTC blank cells and an increase in HER2+ MTCs (Fig. 3c), suggesting that HER2+ MTCs may have a greater propensity to traverse the BBB. A progressive increase in the neuro-related MTC subtype was observed during brain metastasis (Supplementary Fig. 10), indicating potential clonal evolution driven by the brain microenvironment. Meta-module analysis revealed distinct expression patterns for each subtype, except HER2+ (Fig. 3e, Supplementary Fig. 11a) [42]. We identified specific marker genes and associated functions (Fig. 3f, Supplementary Fig. 11b, Supplementary Table 8). MTC blank cells showed high cell motility (e.g., ACTG2) and heightened transcription/translation. Neuro-related MTCs expressed anti-inflammatory genes (e.g., SLP1) and were enriched for cell adhesion. AR+HR+ and HR+ MTCs were characterized by active cell metabolism and resistance to stimuli. HER2+ MTCs retained key breast cancer cell traits (e.g., SCGB2A2) and suppressed the tumor immune response (Fig. 3g). Assessing activity across seven biological processes [16] (Fig. 3h, Supplementary Fig. 12), MTC blank cells exhibited complex inflammatory and stress responses in the bloodstream, while neuro-related MTCs displayed minimal stress signals but highly activated cell development pathways in the brain microenvironment. HER2+ MTCs maintained stable activity in the brain and CSF.

### Developmental trajectory of MTCs during brain metastasis

**Fig. 4:**
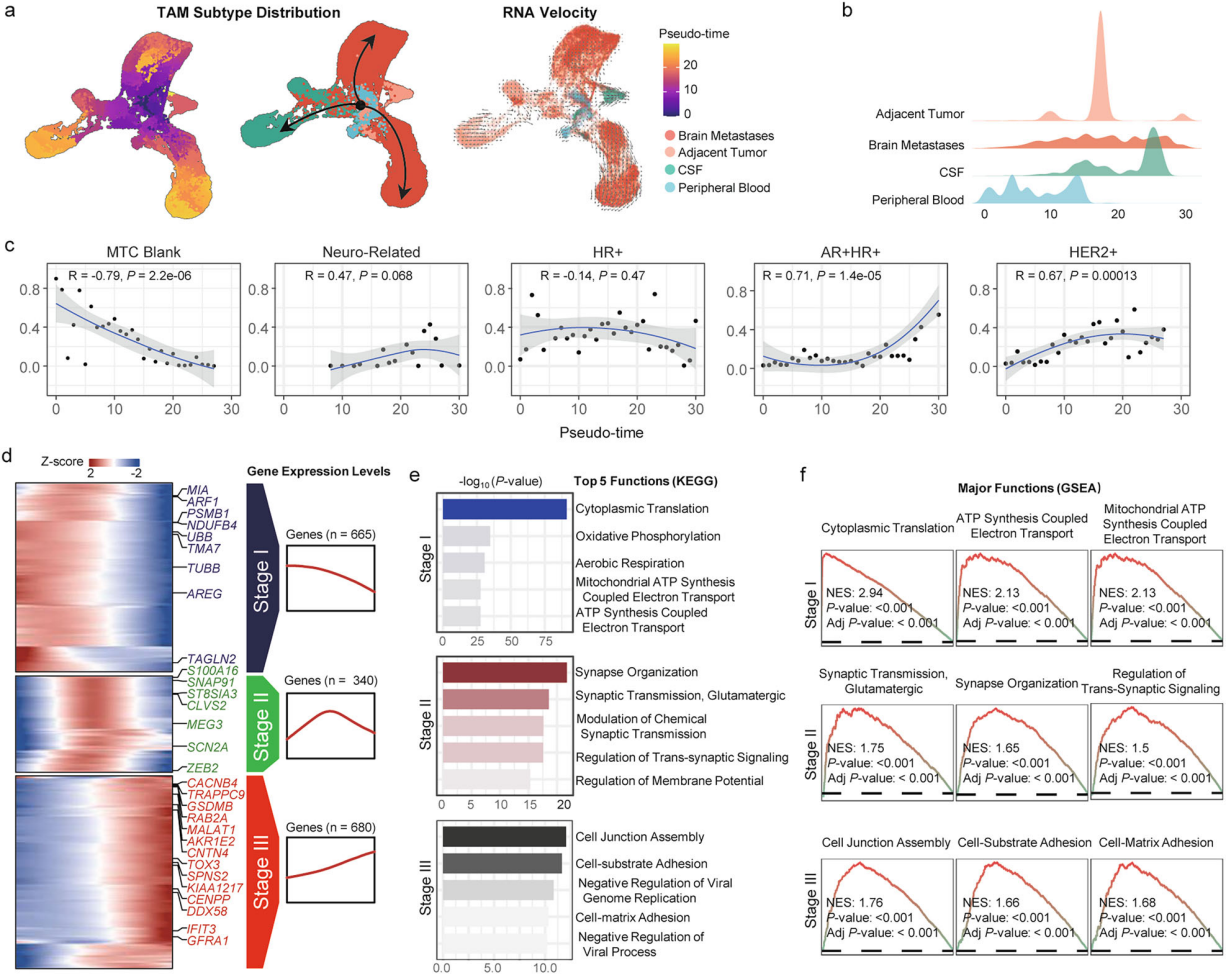
Developmental trajectory of MTCs during brain metastases. **a** Pseudo-time reconstruction and developmental trajectory of MTCs during brain metastases, as inferred by Monocle3. The estimated RNA velocity field, illustrated as streamlines by scVelo, shows the relative RNA states, which align with the developmental trajectories. **b** Ridgeline plots depicting the distribution of cell abundance from different sampling sites across pseudo-time. **c** Scatter plots and correlations of the proportion of the five MTC subtypes in each sample with pseudo-time. **d** Relative expression levels of genes at each of the stages (I–III) of MTC development. **e** Major top-5 biological functions (assessed by KEGG) for stages I–III. **f** Major biological functions (determined by GSEA) for stages I–III.

Pseudo-time and RNA velocity analyses were conducted to uncover the developmental trajectory and potential biological events associated with MTCs crossing the BBB and adapting to the brain microenvironment. These analyses were consistent with a model in which CTCs first traverse the BBB to establish brain metastasis, subsequently infiltrate adjacent tumor tissues, and eventually cross the BFB to enter the CSF (Fig. 4a, b), rather than directly demonstrating each physical transit step. Notably, all MTC subtypes, except the neuro-related subtype, were present at the early stages of the developmental trajectory, supporting the notion that the brain microenvironment induces MTCs to develop neuronal characteristics, which are necessary for the formation of brain metastases (Fig. 4c).

Based on these findings, we categorized MTCs into three developmental stages: Stage I–III, each characterized by distinct marker genes and biological activities. As shown in Fig. 4c, cells with pseudo-time values of 0–10 were assigned to Stage I, 10–20 to Stage II, and greater than 20 to Stage III, reflecting three major transitions along the metastatic progression. In Stage I, MTCs showed highly active energy metabolism and transcription-translation processes, suggesting that they are in a metabolic state consistent with preparation for BBB transit. In Stage II, MTCs activated biological signals and pathways associated with synaptogenesis and neural signaling, indicating that, once within the brain parenchyma, MTCs may “camouflage” themselves by mimicking neural signaling pathways. In Stage III, MTCs predominantly produced signals related to cell adhesion, consistent with mature MTCs infiltrating adjacent tumor tissue and forming dense tumor masses through cell-to-cell adhesive interactions (Fig. 4d–f) (Supplementary Tables 9 and 10). The pseudo-time analysis revealed three critical biological processes central to MTC brain metastasis: amplification of energy metabolism and transcription-translation activity in the early stage, synaptogenesis and neural signaling secretion in the intermediate stage [43], and development of cell adhesion properties in the final stage. These findings provide insight into the dynamic and adaptive nature of MTCs as they evolve to thrive in the brain microenvironment.

### Genotypes of MTCs and CTCs for breast cancer brain metastasis

BCBM presents a significant clinical challenge [44–46]. We initiated an exploratory study aimed at identifying patients at high risk for brain metastasis by analyzing DNA-level alterations. We used inferCNV to analyze copy number variation (CNV) at the single-cell transcriptomic level. Our results showed that all subtypes of MTCs exhibited similar CNV profiles, including amplifications of chromosomes 8, 16, and 21, and deletions of chromosome 13 (Supplementary Fig. 13a–c).

**Fig. 5:**
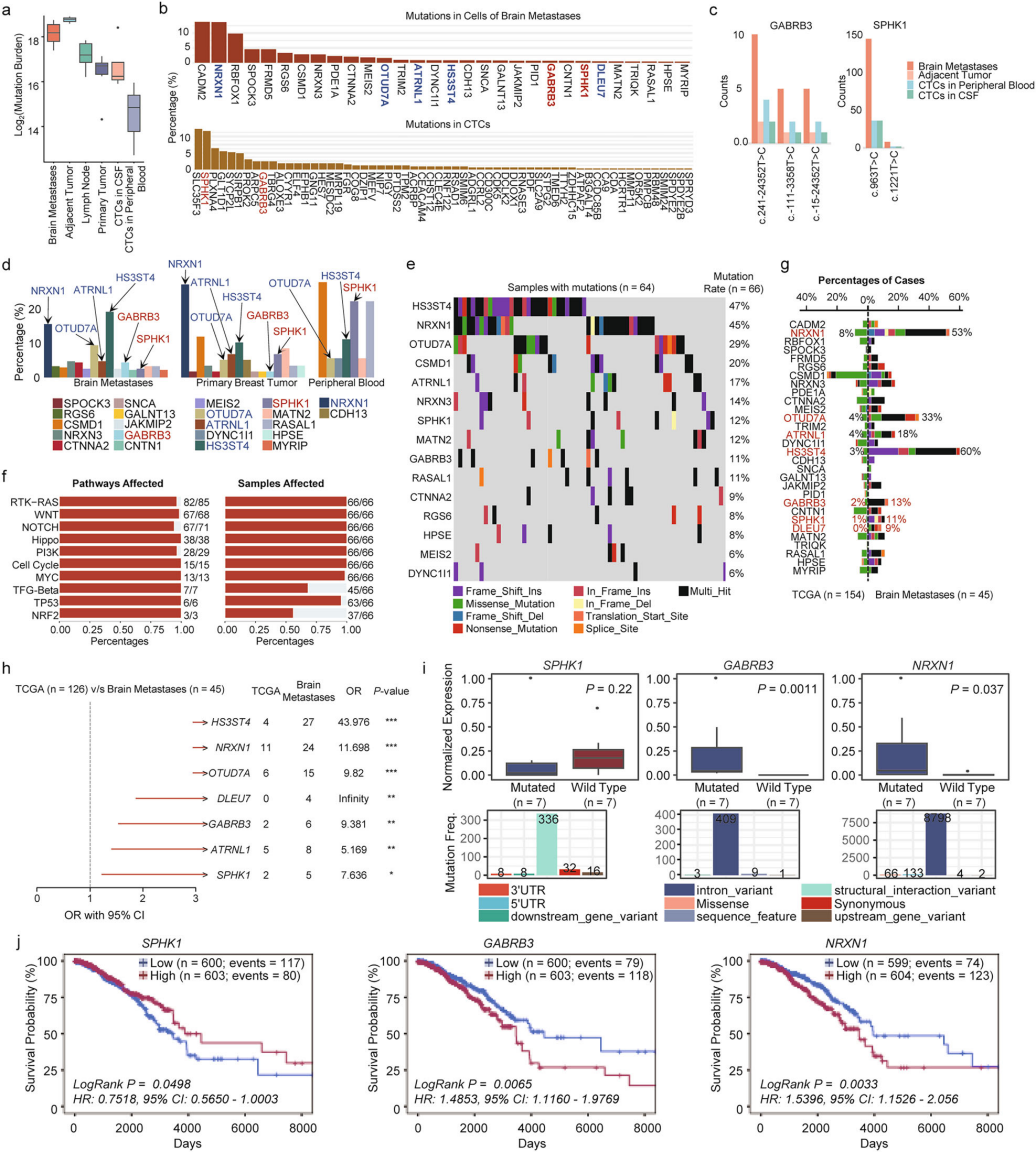
Genomic characteristics of breast cancer brain metastasis in single-cell and bulk-level. **a** Comparison of tumor mutation burden across various tumors, lymph nodes, and CTCs in CSF and peripheral blood. Box plots indicate the median (*center line*), interquartile range (*box bounds*), and highest/lowest values not considered outliers (*whiskers*). **b** Bar plot showing the percentage of the top 30 most mutated genes in brain metastasis cells (*top*) and the percentage of all gene mutations detected in CTCs (*bottom*). Genes mutated in both brain metastasis cells and CTCs are highlighted in red. Genes identified as risk factors for breast cancer brain metastasis (BCBM) are highlighted in blue. **c** Bar plot depicting the count of specific gene mutations in both CTCs and MTCs in brain metastasis. **d** Bar plot showing the percentage of specific gene mutations identified in DNA sequencing. **e** Oncoprint displaying the most frequent oncogenic alterations in all bulk samples. **f** (*Left*) Fractions of cancer-associated pathways affected by the muted genes. (*Right*) Fractions of altered samples per pathway. **g** Co-bar plots comparing the mutation rates of different mutation types across the BCBM and TCGA breast cancer datasets. Genes with statistically higher mutation rates (*P* < 0.05) in BCBM compared to primary breast tumors are highlighted in red. *P*-values were calculated using Fisher’s exact test. **h** Forest plots showing the mutation counts of various mutation types in genes between the TCGA primary breast and BCBM samples. OR represents the odds ratio with 95% CI (OR = 1, no effect; OR > 1, BCBM samples having more mutants than TCGA samples), and *P*-values indicate statistical significance (*P < 0.05; **P < 0.01; ***P < 0.001). **i**. Box plots displaying the normalized RNA expression levels of mutated and wild-type transcripts for the indicated genes in BCBM samples (*n* = 14), as determined by scRNA-seq. The number of brain metastasis samples for each transcript is also indicated. **j** Survival plots correlating the differential expression of the indicated genes with OS in breast cancer patients from the TCGA dataset. Data were retrieved from the UCSC Xena platform and re-visualized using R. High and low expression levels were defined based on the median.

Next, we analyzed gene mutations at single-cell resolution to determine their association with brain metastasis. To strengthen the correlation, we incorporated data from Tokura et al. [47] and the 1000 Genomes Project [48–50]. Brain metastasis tissue exhibited a significantly higher mutational burden than both primary breast tumors and extracranial metastases (Fig. 5a). CTCs in CSF and blood had a lower mutational burden compared to MTCs in tissue samples.

CTCs are critical for metastasis [51–54]. We identified 30 key genes in brain MTCs, with CADM2, NRXN1, and RBFOX1 being the top three most mutated. GABRB3 and SPHK1 were also detected, and their mutations were significantly enriched in brain metastasis-associated CTCs (Fig. 5b, c). These findings suggest GABRB3 and SPHK1 may be key genes for clinical monitoring of BCBM. A comprehensive multi-omics analysis of DNA-seq data (BCBM and TCGA) [55, 56] showed that mutations identified through DNA-seq were consistent with scRNA-seq (Fig. 5d, e), involving pathways like RTK-RAS, WNT, and NOTCH [57–59] (Fig. 5f, Supplementary Fig. 14).

At the bulk tissue level, DNA-seq analysis revealed that the mutation rates of seven genes—NRXN1, OTUD8A, HS3ST4, GABRB3, SPHK1, ATRNL1, and DLEU7—were significantly higher in the BCBM dataset compared with primary breast tumors in the TCGA datasets. Risk analyses indicated that all seven gene mutations were BCBM risk factors (Fig. 5g, h).

In addition to pathway-level genomic alterations, we further characterized the mutation spectrum of GABRB3, NRXN1, and SPHK1 in our DNA-seq dataset. NRXN1 harbored 9,003 mutation entries, almost all corresponding to non-coding regulatory variants, predominantly 3′UTR and upstream/downstream annotations, with no missense, nonsense, frameshift, or splice-site mutations detected. GABRB3 exhibited a similar pattern, with 422 mutation entries also consisting largely of non-coding variants without recurrent protein-altering events. SPHK1 likewise showed predominantly non-coding variants, including 3′UTR and intronic changes.

To assess whether these variants affect transcriptional output, we compared RNA expression levels between mutant and wild-type cells in the scRNA-seq dataset. As shown in Fig. 5i, NRXN1-mutant cells demonstrated significantly elevated transcript expression relative to wild-type cells (*P* = 0.037), and GABRB3-mutant cells similarly exhibited higher expression levels (*P* = 0.0011), suggesting that these non-coding variants may modulate transcriptional regulation or enhancer-associated activity. By contrast, SPHK1-mutant cells did not display increased expression compared to wild-type cells, consistent with the observation that high SPHK1 expression—rather than mutation status—is associated with better OS. Collectively, these findings indicate that non-coding variants in NRXN1 and GABRB3 may contribute to transcriptional upregulation during brain metastatic evolution, whereas SPHK1 expression appears to be regulated independently of its mutation pattern.

### GABRB3 and NRXN1 are highly involved in TAM-MTC communications during brain metastasis

**Fig. 6:**
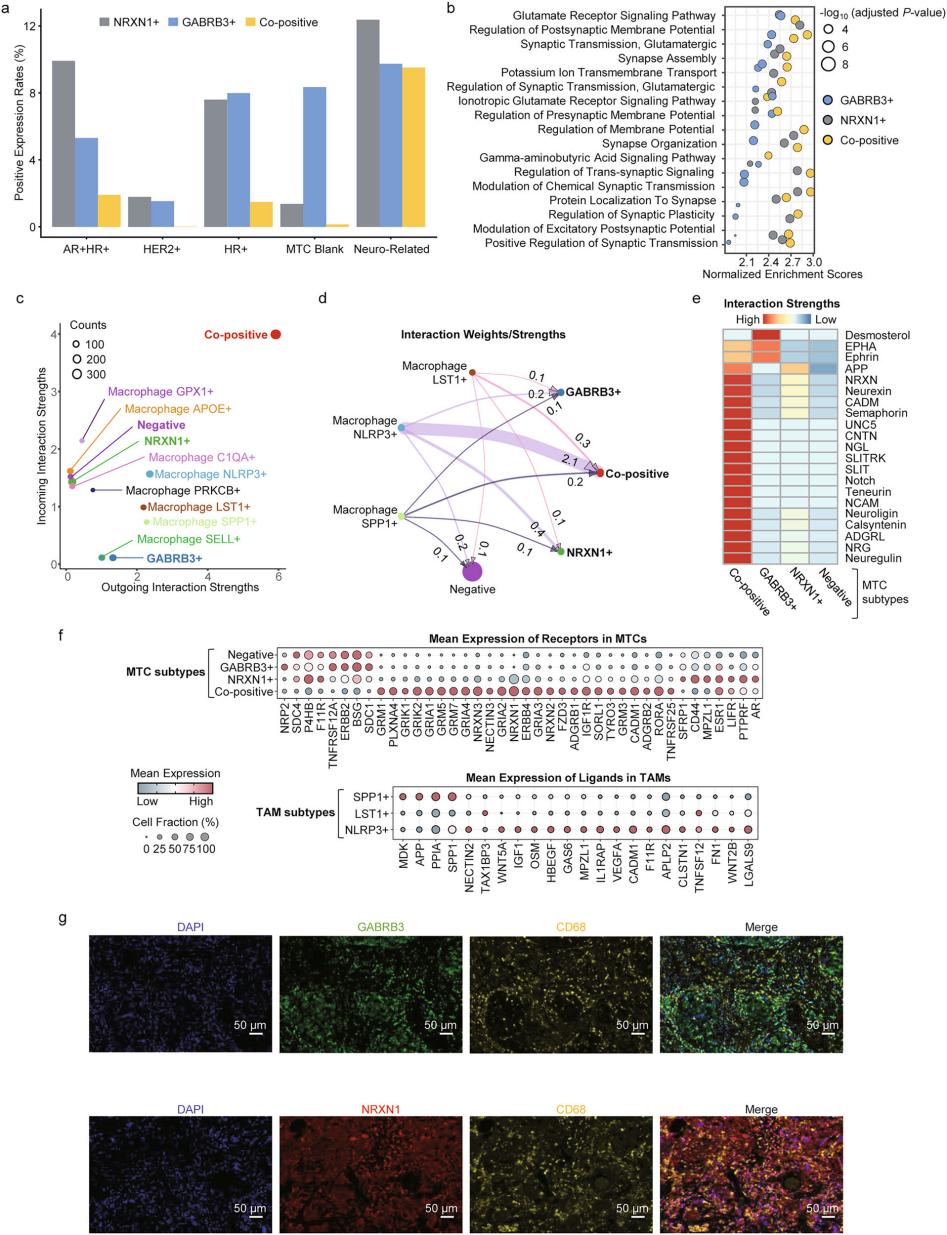
GABRB3 and NRXN1 participate in TAM-MTC communications during brain metastasis. **a** Frequency of *NRXN1* and *GABRB3* expression in the five subtypes of MTCs, as determined by scRNA-seq. **b** GSEA showing neural-related pathways activated by NRXN1 and GABRB3 in cancer cells. **c** Dot plots showing the signal strengths of neural-related cell–cell communications between TAM and MTC subtypes, as analyzed by CellChat. The *x*-axis represents sender cells, and the *y*-axis represents receiver cells. **d** Number of intercellular communications between TAM and MTC subtypes. Line color represents the cell type, and the line thickness corresponds to the number of interactions. **e** Heatmap illustrating neural signaling interactions across cell clusters, showing the correlation between NRXN1 and GABRB3 expression and the strength of neural-related intercellular communication. **f** Ligand-receptor analysis based on CellChat. Dot plots showing the predicted top targets in MTCs (*upper panel*) and ligands in TAMs (*lower panel*). “Negative” refers to the absence of expression of both NRXN1 and GABRB3, while “co-positive” indicates the expression of both NRXN1 and GABRB3. **g** Paraffin-embedded sections of breast cancer brain tissue. Multiplex immunofluorescence staining with antibodies against NRXN1 (red fluorescence), GABRB3 (green fluorescence), CD68 (yellow fluorescence), and DAPI (blue fluorescence), as well as merged images. Scale bar: 50 μm.

Our above findings suggest that the aberrant expression of GABRB3 and NRXN1 may play a key role in driving brain metastasis in breast cancer. We evaluated the expression levels of GABRB3 and NRXN1 across the five molecular subtypes of MTCs. The results demonstrated that GABRB3 and NRXN1 were significantly more positively expressed in HER2-negative MTCs, with co-expression rare except in the neuro-related MTCs (Fig. 6a).

GSEA found that elevated expression of GABRB3 and/or NRXN1 activated neuro-related signaling pathways, particularly those linked to synaptic activity (Fig. 6b). Using the CellChat method, we analyzed neuro-signaling pathways between MTCs expressing GABRB3 and/or NRXN1 and TAMs. As shown in Fig. 6c, NRXN1-positive MTCs primarily act as receivers, and GABRB3-positive MTCs mainly serve as senders. The primary signal senders were NLRP3, LST1, and SPP1 TAMs. The neuro-signaling in NLRP3 TAMs was the strongest, and these signals are most frequently received by NRXN1 and GABRB3-positive MTCs (Fig. 6d). These results indicate that NRXN1 and GABRB3 are likely downstream targets of TAM-associated signaling pathways implicated in brain metastasis colonization. They are highly involved in intercellular communication related to neuronal guidance, neurodevelopment, and neurocellular adhesion processes (Fig. 6e). Ligand-receptor analysis further identified high levels of ligand signaling in NLRP3 TAMs, supporting the possibility that these TAMs may facilitate brain metastasis through growth factor, angiogenesis-related, and neurocellular adhesion-related pathways (Fig. 6f, Supplementary Table 11). To explore whether such signaling could be supported by spatial proximity in situ, we performed multiplex immunofluorescence staining on paraffin-embedded BCBM sections. As shown in Fig. 6g, GABRB3- and NRXN1-positive tumor cells were frequently localized in regions enriched for CD68-positive macrophages, indicating close spatial association between these cell populations.

### Depletion of GABRB3 or NRXN1 suppresses the growth, migration, and invasion of HER2-negative breast cancer cells in vitro and reduces brain metastasis in vivo

**Fig. 7:**
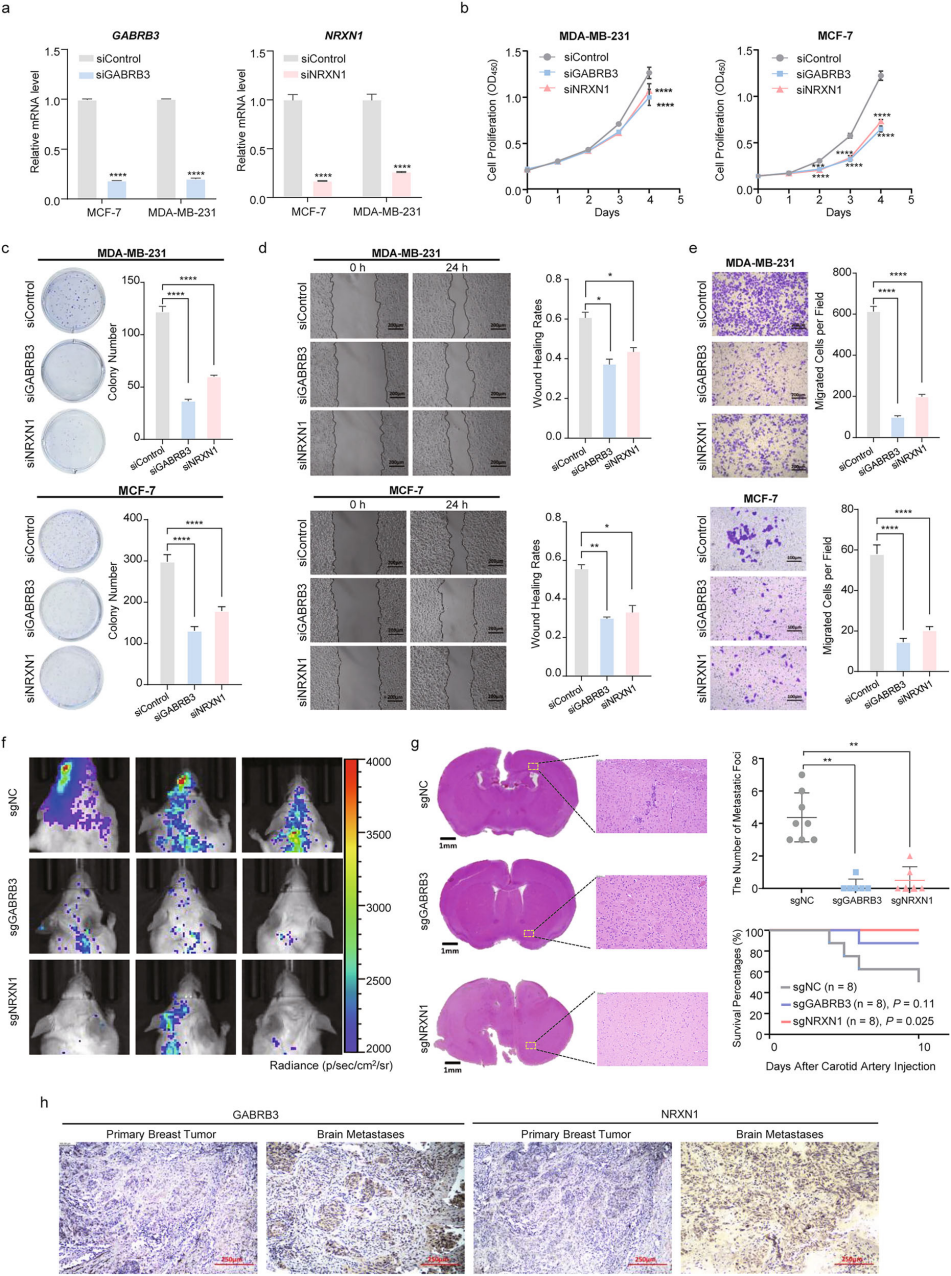
Depletion of GABRB3 or NRXN1 suppresses the growth, migration, invasion, and brain metastasis of HER2-negative breast cancer cells in vitro and in vivo. **a** Human breast cancer cell lines MDA-MB-231 and MCF-7 were transfected with negative control siRNA (siControl) or gene-specific siRNAs targeting *GABRB3* (*siGABRB3*) or *NRXN1*(*siNRXN1*). Knockdown efficiency was verified by RT-qPCR. **b** Cell proliferation was assessed using the CCK-8 assay, with absorbance measured at 450 nm (OD_450_) with a microplate reader. **c** Colony formation assay: Representative images (*left panels*) show colonies in the culture plates. The bar graph (*right panels*) quantifies the colonies. **d** Wound-healing assay: Representative images (*left panels*) show relative cell migration, with the bar graph (*right panels*) indicating the healing rate. **e** Transwell^®^ invasion assay: Representative images (*left panels*) show relative cell invasion. The bar graph (*right panels*) quantifies the invasion. **f** 4T1-LUC cells, stably infected with lentivirus constructs encoding single-guide RNAs for negative control (*sgNC*), *GABRB3* (*sgGABRB3*), or *NRXN1* (*sgNRXN1*), were injected into BALB/c mice (*n* = 8 per group). After 10 days, mice were euthanized, and metastasis was monitored via IVIS imaging. Representative IVIS images are shown. **g** Representative H&E staining of mouse brain tissue illustrates metastatic nodules, which were quantified. Kaplan–Meier OS curves of the mice are also displayed. **h** Representative IHC staining of GABRB3 and NRXN1 proteins in primary breast tumors and unmatched brain metastases. *P < 0.05; **P < 0.01; ****P < 0.0001.

Given the clinical relevance of GABRB3 or NRXN1 expression in breast cancer patients with brain metastasis, as well as their enhanced signaling in HER2-negative MTCs, we investigated their direct impact on the oncogenic properties of HER2-negative breast cancer cells. Using the MDA-MB-231 and MCF-7 cell lines, we knocked down *GABRB3* or *NRXN1* expression by transfecting cells with specific siRNAs targeting these genes (Fig. 7a). Knockdown of either *GABRB3* or *NRXN1* significantly slowed down cell growth compared to control cells (Fig. 7b). Similarly, colony formation assays revealed a marked reduction in colony number following *GABRB3* or *NRXN1* knockdown (Fig. 7c), indicating a suppression of the proliferative capacity of HER2-negative breast cancer cells. On the contrary, overexpression of GABRB3 or NRXN1 in BC cells showed enhanced proliferative capacity (Supplementary Fig. 15a–c; Original Data 1).

To further explore the role of GABRB3 and NRXN1 in cell motility and invasiveness, we performed wound-healing and Transwell^®^ invasion assays. As expected, depletion of GABRB3 or NRXN1 significantly reduced the migration and invasion abilities of both MDA-MB-231 and MCF-7 cells, while overexpression of these genes promoted those of migration and invasion capabilities (Fig. 7d, e and Supplementary Fig. 15d, e). Next, we assessed the impact of GABRB3 and NRXN1 on brain metastasis in a mouse model. *GABRB3* and *NRXN1* genes were stably knocked out in the 4T1-LUC mouse breast cancer cell line using the CRISPR-Cas9 technique (Supplementary Table 9). Stable knockout cells were injected into the left cardiac ventricle of BALB/c mice, and brain metastasis was monitored by IVIS imaging. Depletion of *GABRB3* or *NRXN1* significantly reduced the fluorescent signal in the brain, indicating fewer metastatic foci (Fig. 7f). Histological analysis confirmed the reduced presence of metastases in the brains of mice injected with GABRB3 or NRXN1 knocked out cell lines (Fig. 7g). Furthermore, mice in the *GABRB3* and *NRXN1* knockout groups demonstrated significantly improved OS compared to control mice (Fig. 7g).

To validate these findings in human samples, we detected the expression of the GABRB3 or NRXN1 gene in the tumor regions of brain metastatic tissues and their adjacent normal brain tissues. IHC staining demonstrated that either GABRB3 or NRXN1 was expressed in normal brain tissues, and more importantly, their expression levels were significantly elevated within the tumor regions as compared to the adjacent normal brain tissues. Moreover, consistent with our in vitro and in vivo findings, GABRB3 or NRXN1 exhibited higher expression levels in the brain metastatic tissues of BC patients than in primary breast tissues (Fig. 7h and Supplementary Fig. 15f).

Collectively, our data suggest that GABRB3 and NRXN1 play crucial roles in promoting the growth, migration, and invasion of HER2-negative BC cells in vitro, and contributing to brain metastasis in vivo. Their upregulation might be associated with brain metastatic potentials in BC patients, highlighting the potential of these genes as therapeutic targets in HER2-negative breast cancer.

## Discussion

Our single-cell multi-omics analysis uncovered significant differences in gene expression profiles and biological pathways across various molecular subtypes of MTCs, particularly in their adaptation to the brain microenvironment, including processes such as cell–cell adhesion, immune evasion, and BBB penetration [60, 61]. Previous studies have shown that the interaction between TAMs and microglia is a crucial element of the brain metastasis microenvironment [62, 63]. Targeting TAM–microglia interactions to enhance the efficacy of immunotherapy against intracranial tumors is considered a promising therapeutic strategy. Most TAMs exhibit an M2 phenotype, but reprogramming these M2-type TAMs into M1-type macrophages can reshape the immunosuppressive microenvironment, offering new avenues for cancer treatment [64, 65]. Although the M1–M2 paradigm has dominated TAM research [66, 67], the diverse subtypes of TAMs within tumors complicate its application, highlighting the need for more refined approaches to guide precise treatment approaches [68, 69].

In this study, we performed an in-depth analysis of the BCBM TIME using scRNA-seq, identified a total of twelve macrophage-lineage subtypes, comprising four brain-resident microglial subtypes and eight monocyte-derived TAM subtypes [70]. Our findings show that TAMs display greater heterogeneity compared to microglia and play a pivotal role in brain metastasis. Circulating TAMs exhibit more M1-like characteristics, while tissue-resident TAMs tend to exhibit M2-like features. TAMs demonstrate distinct distribution patterns at different spatiotemporal stages. During the early stages, LST1^+^ macrophages facilitate the crossing of the BBB. At the brain-resident stage, APOE^+^, SPP1^+^, and NLRP3^+^ TAM subtypes help stabilize metastatic foci. Once brain metastases are established, NLRP3 macrophages induce microglia to adopt an M2-like phenotype via multiple signaling pathways. These activated pathways exacerbate local inflammation, suppress effective immune surveillance, and support tumor cell growth and survival [38, 71, 72]. We speculate that disrupting NLRP3⁺ macrophage–microglia interactions or blocking the TGF-β and IL-10 signaling pathways could effectively restore anti-tumor immune responses and inhibit tumor growth in the brain. Beyond intrinsic genetic alterations, the brain TIME likely provides extrinsic cues that sustain these neuro-related programs. Our ligand-receptor analysis suggests a potential signaling axis where NLRP3+ TAMs serve as major signal senders, secreting growth factors such as IGF-1 that converge on metastatic tumor cells (MTCs). Mechanistically, IGF1-IGF1R signaling is known to activate PI3K-AKT cascades, which in turn modulate transcription factors such as FOXO1 and STAT3 [69, 73]. Notably, STAT3 activation has been implicated in the transcriptional regulation of genes associated with neuronal differentiation and microenvironmental adaptation. We propose that these microenvironmental signals may synergize with non-coding mutations to robustly upregulate NRXN1 and GABRB3, facilitating the survival of MTCs in the brain niche.

Our analysis revealed that MTCs can be classified into five main molecular subtypes. MTC progression can be broadly categorized into three stages, with the mid-stage marked by synaptogenesis and neuronal signaling. Notably, neuro-related MTCs interact with the intracranial TIME to activate genes linked to neuronal development, enhancing their adaptability within the CNS—traits absent in primary breast tumors. This adaptive process, facilitated by brain microenvironment signals, is likely to be critical for BBB traversal and subsequent colonization [74, 75]. The functional significance of this upregulation lies in neural mimicry. NRXN1 encodes a synaptic adhesion molecule, while GABRB3 is a key subunit of the GABA-A receptor complex. By co-opting these neuronal signaling programs, MTCs may enhance their synaptic-like interactions with the brain parenchyma, effectively hijacking endogenous pathways for metastatic colonization [74, 75]. Our functional assays demonstrate that depletion of NRXN1 or GABRB3 significantly impairs tumor growth and brain metastasis formation, further underlining their role as critical mediators of this adaptive process.

Research has demonstrated that primary tumor sites and brain metastases exhibit genomic and transcriptomic heterogeneity, although different brain metastatic sites often share high genetic homogeneity [13]. These insights suggest that a small number of clonal subtypes with an innate propensity for brain metastasis may be present in the primary tumor [76]. In our study, the identification of NRXN1 and GABRB3 as key candidates in these subclones is particularly intriguing, given that the majority of these mutations were non-coding regulatory variants. Emerging evidence suggests that such alterations can reshape enhancer activity, alter transcription factor binding motifs, or modulate chromatin accessibility, thereby increasing transcriptional output without altering protein sequences [77, 78]. The observed elevation of NRXN1 and GABRB3 transcripts in mutant cells supports a hypothesis of cis-regulatory activation, potentially via enhancer reprogramming or three-dimensional chromatin looping [79]. Ultimately, these non-coding variants may function as critical regulatory switches that prime tumor cells for brain-specific adaptation, driving their survival and colonization in the brain niche.

Several limitations of this study should be acknowledged. First, our cohort is relatively small and predominantly female (six women and one man), and the single-cell atlas we present is therefore most directly generalizable to female BCBM. Whether the same TAM and MTC programs, as well as GABRB3/NRXN1-associated features, are fully shared in male breast cancer or in larger HER2-negative series remains to be determined. Second, our analyses are primarily observational and integrate single-cell, bulk DNA-seq, and public datasets rather than independent prospective validation cohorts. As a result, the associations we report between mutational burden, CNV patterns, cell-state programs, and clinical outcomes should be interpreted as hypothesis-generating and will require confirmation in larger, multi-institutional studies.

In conclusion, our research leverages single-cell multi-omics approaches to unravel the complex biological characteristics of BCBM, highlights GABRB3 and NRXN1 as key candidates associated with the brain metastasis process, and underscores the significant contribution of TAMs in shaping the brain microenvironment and facilitating tumor adaptation.

## Supplementary information

Supplementary Methods

Supplementary Figures 1–15

Supplementary Information Summary

Supplementary Table 1

Supplementary Table 2

Supplementary Table 3

Supplementary Table 4

Supplementary Table 5

Supplementary Table 6

Supplementary Table 7

Supplementary Table 8

Supplementary Table 9

Supplementary Table10

Supplementary Table 11

Supplementary Table 12

Original Data

## Data Availability

All datasets, data processing codes, and configuration files are available on GitHub (https://github.com/yuansh3354/scBRCA_BM). The scRNA-seq data generated in this study are accessible via the China National Center for Bioinformation (BioProject ID: PRJCA020509, Accession Number: HRA008291). High-resolution images and clinical data have also been deposited in the GitHub repository. Additional public datasets utilized in this study include the human reference genome from 10x Genomics (https://www.10xgenomics.com/support/software/cell-ranger/downloads, refdata-gex-GRCh38-2020-A), TCGA datasets (https://xenabrowser.net/), and scRNA-seq datasets available in the GEO database (Accession Numbers: GSE143423, GSE150660, GSE195861, GSE186344, GSE202501). Source data are provided with this publication.
