# Supplementary material for: Single-cell profiling reveals distinct populations of tumor-associated macrophages and metastatic tumor cells in breast cancer brain metastasis
Source: Cell Death Dis. 2026 Apr 25;17(1):553. doi: 10.1038/s41419-026-08807-w (PMC13247122; doi:10.1038/s41419-026-08807-w)
Supplement: Supplementary file 1 — Supplementary Methods [file 41419_2026_8807_MOESM1_ESM.docx]

**Supplementary Methods**

**Magnetic resonance imaging acquisition**

Brain magnetic resonance imaging (MRI) was performed on all patients within one week prior to surgery on either a 3 Tesla scanner (MR-750, General Electric, USA) or a 1.5 Tesla scanner (Prodiva, Philips, Netherlands). The imaging protocol included T1-weighted and contrast-enhanced T1-weighted sequences (following the administration of a 0.1 ml/kg dose of gadoterate meglumine (337mg/ml, Guerbet, France), as well as T2-weighted, T2 fluid-attenuated inversion recovery (FLAIR), and diffusion-weighted imaging (DWI) using b-values of 0 and 1000. An apparent diffusion coefficient (ADC) map was generated from the b1000 data. MRI scans performed at external institutions were accepted if they met quality and protocol standards. All mages were independently reviewed by two radiologists with over 5 years of experience in brain MRI interpretation. A final review was conducted by a senior radiologist with more than 20 years of experience in brain MRI analysis. Tumour volumes were manually delineated using ITK-SNAP software ([www.itksnap.org](http://www.itksnap.org)).

**Histologic analysis**

All tumor samples were sectioned at 4μm from formalin-fixed paraffin-embedded (FFPE) blocks and stained with H&E as well as IHC antibodies. The IHC panel included, but was not limited to, antibodies for estrogen receptor (ER; clone EP1, 1:100, OriGene), progesterone receptor (PR; clone EP2, 1:100, OriGene), Ki-67 (clone MIB1, 1:100, OriGene), and HER2 (clone 4B5, Ventana).

**Definitions and assessment criteria of HER2 and hormone receptors in breast cancer pathology**

HER2 positive is defined as an IHC membrane staining score of 3+. HER2 negativity includes IHC scores of 1+ and 0. For cases with an equivocal HER2 IHC score of 2+, fluorescence in situ hybridization (FISH) testing is conducted. According to the ASCO/CAP guidelines, HER2 positivity by FISH is determined by a HER2/CEP17 ratio of ≥ 2.0 or a HER2 gene copy number of ≥ 6. ER and PR status were also assessed by IHC, with a positivity threshold set at ≥ 1%. Hormone receptor (HR) positivity was defined as ER-positive and/or PR-positive, while HR negativity was classified as both ER-negative and PR-negative.

**Protocol of CTCs isolation and enrichment from blood**

(1) Collect 10 mL of the peripheral blood sample into a 15 mL centrifuge tube. (2) Add 50 µL/mL of antibody mix to the blood sample. Mix gently and allow it to stand at room temperature for 5 minutes. (3) Vortex the magnetic beads for 30 seconds to ensure even dispersion. (4) Add 50 µL/mL of magnetic beads to the blood sample and mix thoroughly. (5) Prepare the diluent of PBS with 2% fetal bovine serum (FBS) and 1 mM EDTA, free of Ca²⁺ and Mg²⁺. Add an equal volume of diluent to the blood sample to double its volume. Mix gently by pipetting up and down 2-3 times. (6) Place the centrifuge tube (with the cap removed) in a magnetic rack and incubate for 10 minutes at room temperature. (7) Transfer the supernatant a new centrifuge tube. (8) Add the magnetic beads to the sample in the same volume as in step 4. (9) Incubate the tube (without cap) in the magnetic rack for 10 minutes at room temperature. (10) Carefully transfer the enriched cell suspension to a new tube. (11) Collect the enriched cells for further analysis. (12) Transport the enriched cells to Berry Genomics Corporation (Beijing, China) within 1 hour for library construction and sequencing.

The reagents used: FBS (Gibco, USA); FITC anti-human CD326 (Ep-CAM), and Alexa Fluor^®^ 647 anti-human CD45 antibodies (Biolegend, USA); Hoechst 33342 (Sigma-Aldrich, USA); Lymphocyte Isolate, RosetteSep™ Human CD45 Depletion Cocktail, RosetteSep™ CTC Enrichment Cocktail Containing Anti-CD36, RosetteSep™ CTC Enrichment Cocktail Containing Anti-CD56, and Alexa Fluor^®^ 647 anti-human CD45 antibodies (Biolegend, USA); RosetteSep™ CTC Enrichment Cocktail Containing Anti-CD36, RosetteSep™ CTC Enrichment Cocktail Containing Anti-CD56, and EasySep™ Direct Human CTC Enrichment Kit (STEMCELL, Canada).

**scRNA-seq library preparations and data pre-processing**

Both library construction and sequencing were carried out at the Berry Genomics Corporation. Fresh tissue samples were maintained in cold MACS storage solution (2–4 °C; No. 130-100-008) immediately after surgery and then cut into approximately 0.5–1 g pieces. Samples were transported to the Berry Genomics Corporation (Beijing, China) within 1–3 hours for tissue dissociation and subsequent sequencing on the Illumina NovaSeq 6000 platform (Illumina, USA). For all samples, scRNA-seq data were aligned and quantified using the CellRanger toolkit (Version 7.1.0) against the GRCh38 reference genome (10x/refdata-gex-GRCh38-2020-A). Quality assessment of the cells was based on the following metrics: (1) total UMI counts per cell (library size) below 60,000; (2) detection of more than 200 but fewer than 10,000 genes per cell; (3) mitochondrial gene content under 20%; and (4) features detected in at least 100 cells. We achieved a median of 5,374 cells per sample, with an average of 13,001 read counts and a median of 2,879 genes detected per cell.

In addition to these standard quality filters, we applied two additional QC procedures to further ensure data accuracy. Doublets were identified and removed using DoubletFinder (version 2.0), with the expected doublet rate estimated from CellRanger for each library. Parameter optimization was performed per sample (pN = 0.25; pK determined through paramSweep and bcmvn), and cells with a predicted doublet score above the sample-specific threshold were excluded. Across all samples, approximately 3.8% of cells were removed as predicted doublets. Ambient RNA contamination was corrected using SoupX (version 1.6.0), which estimates contamination fractions from empty droplets and generates corrected expression matrices for downstream analysis. The inferred contamination fractions ranged from 2% to 8% across samples.

After doublet removal, ambient RNA correction, and initial QC filtering, a total of 131,880 high-quality cells were retained for downstream analysis. To integrate data from multiple sources and clinical sites, we performed batch correction using the Harmony algorithm (v1.2.0) on the PCA space, with individual samples treated as the batch variable. All parameters were set to their default values as implemented in the Harmony package. This approach allowed for the identification of shared cell states across the cohort while effectively minimizing patient-specific technical noise and preserving biological heterogeneity.

**Library construction and sequencing**

scRNA-seq libraries were prepared using 10,000 cells per sample. The Chromium Single cell 3' Library and Gel Bead Kit Version 3.1 (10× Genomics, PN1000268) was employed to generate single-cell gel beads in emulsion (GEM). Captured cells were lysed, and the released RNA was reverse-transcribed with primers containing poly-T, barcode, unique molecular identifiers (UMIs) and the Read 1 primer sequence. The barcoded cDNA was then purified and amplified by PCR. The adapter ligation step was performed to incorporate sample indices and the Read 2 primer sequence. Following quality control, the libraries were sequenced on Illumina Novaseq 6000 platform using 150 bp pair-ended reads (The Berry Genomics Corporation, Beijing, China).

**Cell annotation**

The SCTransform function was applied with the glmGamPoi method to reduce technical variability, regressing out nFeature_RNA (method = "glmGamPoi", vars.to.regress = "nFeature_RNA"). Principal component analysis (PCA) was performed using the top 50 PCs calculated with RunPCA function. We then applied UMAP dimensionality reduction on the scaled matrix using the top 30 PCA components to generate a two-dimensional representation. For clustering, we used the Seurat's FindClusters function (Seurat::FindClusters), which implements a shared nearest neighbor (SNN) modularity optimization algorithm, setting the resolution parameter to 2 on the top 30 PCA components.

Based on known marker genes, we annotated nine major cells types in our dataset as follows: EPCAM, SCGB2A2, CD24, KRT7, KRT8, KRT18 and KRT19 for MTCs; CSF3R, S100A8 and S100A9 for Neutrophils; AIF1, LYZ and CD163 for Macrophages; TMEM119, CSF1R and ITGAM for Microglia; CD3D, CD3E and CD2 for T Cells; RGS5, TAGLN and ACTA2 for Vascular Smooth Muscle; CLDN5, PECAM1 and RAMP2 for Endothelial Cells; GFAP, SLC1A2 and AQP4 for Astrocytes; MBP, MOBP and MOG for Oligodendrocytes. Microglia were identified using the canonical CNS-resident markers TMEM119, P2RY12, and SALL1, together with homeostatic genes CX3CR1, CSF1R, and OLFML3. Monocyte-derived macrophages (TAMs) were annotated by expression of LYZ, CD163, AIF1, LST1, SPP1, APOE, and NLRP3, representing peripheral inflammatory and immunosuppressive programs.

**Copy number variation analysis**

Copy number variation (CNV) analysis was conducted scRNA-seq data using the inferCNV R package (Version 1.16.0) (https://github.com/broadinstitute/inferCNV). MTCs were designated as observation cells, while all other cell types served as reference cells. A minimum average read count threshold of 0.1 per gene across reference cells was applied. Due to the large data volume in this study, inferCNV could not process all cells simultaneously. As a solution, inferCNV was executed separately for data generated in this study and for data obtained from public databases. To ensure comparability, the same reference cells were consistently used across both datasets.

**Computation of cluster heterogeneity**

To assess heterogeneity, we extracted the count matrix for all MTCs of different molecular types from tumor tissues, aiming to minimize the influence of sample sources variability. Each molecular type was represented as a separate Seurat object. Using the top 5 principal components (PCs), UMAP dimensionality reduction was applied to each Seurat object. Clustering for each molecular type was performed with Seurat's FindClusters function, using default parameter settings. For each cluster, we computed the geometric center and then calculated the Euclidean distance of each MTC in the cluster from its geometric center. The sum of these distances provided the heterogeneity score for each cluster.

**Pathway enrichment analyses**

Gene Set Enrichment Analysis (GSEA): Pre-ranked gene lists, ordered by avg_log2FC values obtained from Searat::FindAllMarkers, were used as input for GSEA. This analysis was performed with the GseaVis::gseaNb function from the GseaVis R package (version 0.0.8) (https://github.com/junjunlab/).

Kyoto Encyclopedia of Genes and Genomes (KEGG) Pathway Enrichment Analysis: KEGG pathway enrichment was conducted using the ClusterGVis::enrichCluster function from the ClusterGVis R package(version 0.1.0), with gene annotations from org.Hs.eg.db (version 3.17.0).

For visualization in radar plots, pathway activity scores (e.g., GSEA or KEGG module scores) were first min–max normalized within each panel to the [0, 1] range and then linearly rescaled to 0–100% for display. Each radial axis represents a distinct pathway or functional category, and the distance from the center corresponds to the normalized score of that pathway for the indicated cell subtype. For butterfly plots, each axis represents the relative meta-module score of one MTC molecular subtype. Cells were positioned in the two-dimensional space according to their standardized (z-scored) signature scores, such that the distance from each axis reflects the relative contribution of the corresponding subtype-specific signature. Unless otherwise specified, all statistical tests reported in this study were two-sided. For differential expression and module-score comparisons between groups (e.g., resident versus circulating TAMs, or between MTC subtypes), we used the Wilcoxon rank-sum test as implemented in Seurat. Multiple testing correction was performed using the Benjamini–Hochberg false discovery rate (FDR) procedure, and adjusted P-values (FDR < 0.05) were used to define statistical significance in enrichment analyses and cell–cell communication tests.

**Single-cell level somatic mutations calling**

Cell-Level Analysis: Somatic mutation analysis at the single-cell level was conducted using the Numbat R package (version 1.3.2). SNP pileup data were generated with cellsnp-lite, while phasing was performed with eagle2. High-polymorphic regions were identified using WGS data from the 1000 Genomes Project, mapped to the GRCh38 reference genome. Numbat was then used to call somatic mutations and produce VCF files for downstream analysis, with parameters and configurations set according to the Numbat documentation. The resulting VCF files were annotated using the snpEff software (version 4.3t build 2017-11-24), which also employed the GRCh38 reference genome.

Cell Type-Level Analysis: First, we annotate the cell type of each MTC generating a list with cell barcodes in the first column and cell identities in the second. BAM files from the scRNA-seq data then split based on cell identity, as specified in this list. SComatic was used to perform the mutation calling, producing VCF files for further analysis. All parameters, configurations, and reference data were set to the default values or files provided by SComatic. The resulting VCF files were annotated using the snpEff software (version 4.3t build 2017-11-24), with the GRCh38 reference genome.

**Bulk DNA-seq analysis**

A total of 200 ng to 1 μg DNA per sample was fragmented to approximately 300 bp using a Covaris S2 ultrasonicator. Library preparation was conducted with the NEBNext Ultra DNA Library Prep Kit for Illumina, followed by post-hybridization amplification, quality checks, and sequencing, which was performed by the Precision Scientific (Beijing) Co.,Ltd. (Beijing, China). Quality control of the raw FASTQ data was performed with FastQC (Version 0.12.1), and quality reports were compiled using MultiQC (Version 1.13). Low-quality reads (below Q25) were filtered out using trim_galore (Version 0.6.10). The resulting high-quality reads were aligned to the human reference genome GRCh38 using BWA-MEM (version 0.7.17-r1188). Further data processing and variant calling were conducted with The Genome Analysis Toolkit (GATK, version 4.3.0.0), HTSJDK (version 3.0.1) and Picard (Version 2.27.5). The main reference datasets for GATK analysis included: dbsnp_146.hg38.vcf.gz, 1000G_phase1.snps.high_confidence.hg38.vcf.gz, Mills_and_1000G_gold_standard. indels. hg38.vcf.gz, and af-only-gnomad.hg38.vcf.gz, among others.

**Developmental trajectory and RNA velocity analysis**

To infer developmental trajectories of MTCs, we used Monocle3 (version 1.4.1) with explicitly defined dimensionality reduction, graph construction, and pseudotime ordering parameters. The preprocessed Seurat object was converted to a cell_data_set using as.cell_data_set, followed by dimensionality reduction using UMAP with 30 principal components (reduce_dimension(reduction_method = "UMAP", preprocess_method = "PCA", umap.min_dist = 0.1)). To account for potential batch effects during trajectory construction, we utilized the align_cds function with default parameters to harmonize data across different samples. Trajectory graph learning was performed using learn_graph(use_partition = TRUE, close_loop = FALSE, learn_graph_control = list(prune_graph = TRUE)), allowing Monocle3 to construct a principal graph that captures the dominant developmental continuum of MTCs. Cells were ordered along pseudotime using order_cells. The root state was programmatically assigned to the peripheral blood-derived cell population, based on the biological premise that circulating myeloid cells serve as the primary developmental precursors to the monocyte-derived TAMs that colonize the brain metastatic niche. This initialization ensures that the trajectory reflects the physiological recruitment and adaptation process, which was further cross-validated by the earliest-direction cell group inferred from scVelo latent time. This procedure yields a continuous pseudotime spanning Stage I–Stage III, reflecting successive transcriptional transitions during metastatic colonization.

For RNA velocity analysis, we used scVelo (version 0.2) under the dynamical model, which allows inference of transcriptional kinetics by modeling splicing dynamics. Raw spliced and unspliced matrices were first processed using scvelo.pp.filter_and_normalize(min_counts = 3, min_cells = 20, n_top_genes = 2000) to select high-quality velocity-informative genes. Moments were computed with 30 nearest neighbors and 30 PCs using scvelo.pp.moments(n_neighbors = 30, n_pcs = 30). RNA velocity was then estimated using scvelo.tl.velocity(mode = "dynamical"), and embedding-level velocities were visualized with scvelo.tl.velocity_graph. Latent time was computed using scvelo.tl.latent_time, providing an orthogonal temporal measure that was consistent with Monocle3 pseudotime ordering. Together, these settings satisfy the assumptions of the scVelo dynamical framework, namely that transcription, splicing, and degradation follow first-order kinetics and that splicing states can be used to infer transcriptional directionality.

**Cell-cell communication analysis**

Cell–cell communication analysis was conducted on the scRNA-seq data using the CellPhoneDB software (version 3.0.0). Only receptors and ligands expressed in more than 1% of cells were included in the analysis, and interactions were considered absent if either the ligand or the receptor was unmeasurable. For each ligand-receptor pair, the average expression was calculated across different cell types Only pairs with a P-value < 0. 05 were retained to predict between cell types.

**Cell culture and transfections, plasmids, lentivirus production, RNA oligonucleotides and Reagents**

Human breast cancer cell lines MDA-MB-231 and MCF-7 were cultured in DMEM high-glucose medium (Gibco, Cat#: 11965092) supplemented with 10% fetal bovine serum (EVERY GREEN, Cat#: 11011-8611) and 1% penicillin–streptomycin (Gibco, Cat#: 15140122). The mouse breast cancer cell line 4T1-luc were maintained in RPIM-1640 medium (Gibco, Cat#: 11875093) supplemented with 10% fetal bovine serum and 1% penicillin–streptomycin. All cell lines were incubated at 37 °C in a humidified incubator with 5% CO2.

Target siRNA and sgRNA sequences for GABRB3 and NRXN1 (from JTS Scientific) are provided in Table S1. To construct lentiviral vectors for GABRB3 and NRXN1 sgRNA, these sequences were cloned into the lentiCRISPR-V2 (MIAOLING Biology). Lentiviruses were produced by co- transfecting HEK293T cells with recombinant lentivirus vectors and packaging plasmids, psPAX2 and pMD2G (MIAOLING Biology). Stable cell lines were selected using puromycin, and single cell clones were sorted into 96-well plates for clonal expansion. Gene editing for GABRB3 and NRXN1 was confirmed by Sanger sequencing to identify indels.

Transfections of siRNAs were performed with Lipofectamine RNAi MAX (Invitrogen, Cat#: 13778150) according to the manufacturer’s instructions. Anti-NRXN1 antibody (Rabbit Polyclonal, Cat#: PS12916S) and anti-GABRB3 antibody (Rabbit Polyclonal, Cat#: PH19148S) were purchased from ABMART.

**Reverse transcription-quantitative polymerase chain reaction (RT-qPCR)**

Total RNA was isolated from cells using TRIzol^®^ reagent (Thermo Fisher Scientific, Cat#: 15596026CN) following the manufacturer’s instructions. Reverse transcription was conducted using the PrimeScript™ RT reagent kit (Takara, Cat#: 6210a) as per the provided protocol. Quantitative PCR (qPCR) was then performed using TB Green^®^ Premix Ex Taq™ II (TAKARA, Cat#: RR820Q) on a StepOnePlus™ Real-Time PCR. GAPDH was used as the internal control.

**Cell growth and colony formation assay**

Cell proliferation was assessed using the Cell Counting Kit-8 (CCK-8; Beyotime, Cat#: C0037). At designated time points (1, 3, 5, and 7 days), 10 μL of CCK-8 solution and 90 μL of DMEM medium containing 10% FBS was added to each well. After a 2-hour incubation, absorbance was measured at 450 nm. For the colony formation assay, cells were seeded at 500 cells/well in 6-well plates and cultured in DMEM medium supplemented with 10% FBS at 37 °C for 10 days. Colonies were fixed with 4% paraformaldehyde, stained with crystal violet (Beyotime, Cat#: C0121), and counted. All experiments were conducted in triplicate.

**Cell migration and invasion analysis**

Cell migration was assessed using a wound healing assay. Cells were seeded in 12-well plates at a density of 200,000 cells/well and cultured in DMEM medium supplemented with 10% FBS at 37 °C for 24 h until reaching approximately 90% confluency. A scratch was introduced, and wound healing rates were measured by comparing the scratch width at each time point to the initial width at 0 hours.

For the invasion assay, cells were seeded in 6.5 mm Transwell^®^ chambers with 8.0 µm pore polycarbonate membrane insert Chambers (Corning, Cat#: 3422) and incubated in a 5% CO2 atmosphere at 37 °C. After 24 hours, cells that had invaded to the underside of the membrane were fixed with 4% paraformaldehyde and stained with 0.5% crystal violet for 30 minutes, and counted under a microscope. Images were captured to document invasive cells at the bottom of the chamber.

**Animal experiments**

All animal experiments were approved and guided by the Ethics Committee of the Beijing Institute of Biotechnology, with mice housed in specific pathogen–free (SPF) conditions. Female BALB/c nude mice (6 weeks old, 18-22 g) were obtained from Beijing SIPEIFU Biotechnology Company. Brain metastasis imaging was performed using the IVIS SpectrumCT bioluminescence imaging system (PerkinElmer).

For in vivo imaging, 24 six-week-old female BALB/c mice were randomly divided into three groups (8 mice per group) and injected with 5 × 10^5^ sgControl 4T1-luc cells, sgABRB3 4T1-luc cells, or sgNRXN1 4T1-luc cells via intracardiac injection. Following the principle of complete randomization, each mouse received an injection into the left cardiac ventricle. Ten days post-injection, D-luciferin (200 mg/kg) was administered intraperitoneally, and mice were imaged 15 minutes later. During imaging, mice were positioned on a warmed stage in the camera box and continuously sedated with 2.5% isoflurane. Each group was imaged for 5 minutes, with bioluminescent signals captured, integrated, and digitized by the IVIS camera system.

To assess brain metastases, brain tissues were harvested at necropsy, fixed in 4% formaldehyde, and embedded in paraffin blocks. Tissue sections were then stained with hematoxylin and eosin (H&E) for histological evaluation.

**Immunohistochemistry**

For IHC analysis, tissue sections were deparaffinized, rehydrated, and treated with 3% H2O2 for 15 minutes to block endogenous peroxidase activity. Antigen retrieval was performed by heating sections in 10 mM citrate buffer (pH 6.0) in a microwaved for 30 minutes. Tissue sections were then incubated overnight at 4°C with primary antibodies (anti-GABRB3 and anti-NRXN1) at a 1:100 ratio dilution. Subsequent steps were carried out following the instructions provided in the IHC staining kit (ZSGB-BIO, Cat#: SPN-9001). Finally, 3,3’-diaminobenzidine (DAB) tetrachloride hydrate was applied as the chromogen to visualize the signal.

For quantitative assessment of GABRB3 and NRXN1 protein expression, staining intensity was evaluated using a semi-quantitative H-score system ranging from 0 to 300. Two board-certified pathologists, blinded to clinical and molecular information, independently estimated the percentage of tumor cells showing weak (1+), moderate (2+), or strong (3+) cytoplasmic and/or membranous staining in each section. The H-score for each case was calculated as: H-score = (percentage of 1+ cells × 1) + (percentage of 2+ cells × 2) + (percentage of 3+ cells × 3). For downstream analyses, primary breast tumors and brain metastases were unmatched (i.e., not paired from the same individual). Thus, comparisons of continuous H-scores between primary and metastatic lesions were performed using two-sided Mann–Whitney U tests. When dichotomizing staining into “high” and “low” categories, the median H-score within the IHC cohort was used as the positivity threshold, and group differences were evaluated using two-sided Fisher’s exact tests.
