# Supplementary material for: Single-cell profiling reveals distinct populations of tumor-associated macrophages and metastatic tumor cells in breast cancer brain metastasis
Source: Cell Death Dis. 2026 Apr 25;17(1):553. doi: 10.1038/s41419-026-08807-w (PMC13247122; doi:10.1038/s41419-026-08807-w)
Supplement: Supplementary file 2 — Supplementary Figures 1–15 [file 41419_2026_8807_MOESM2_ESM.pdf]

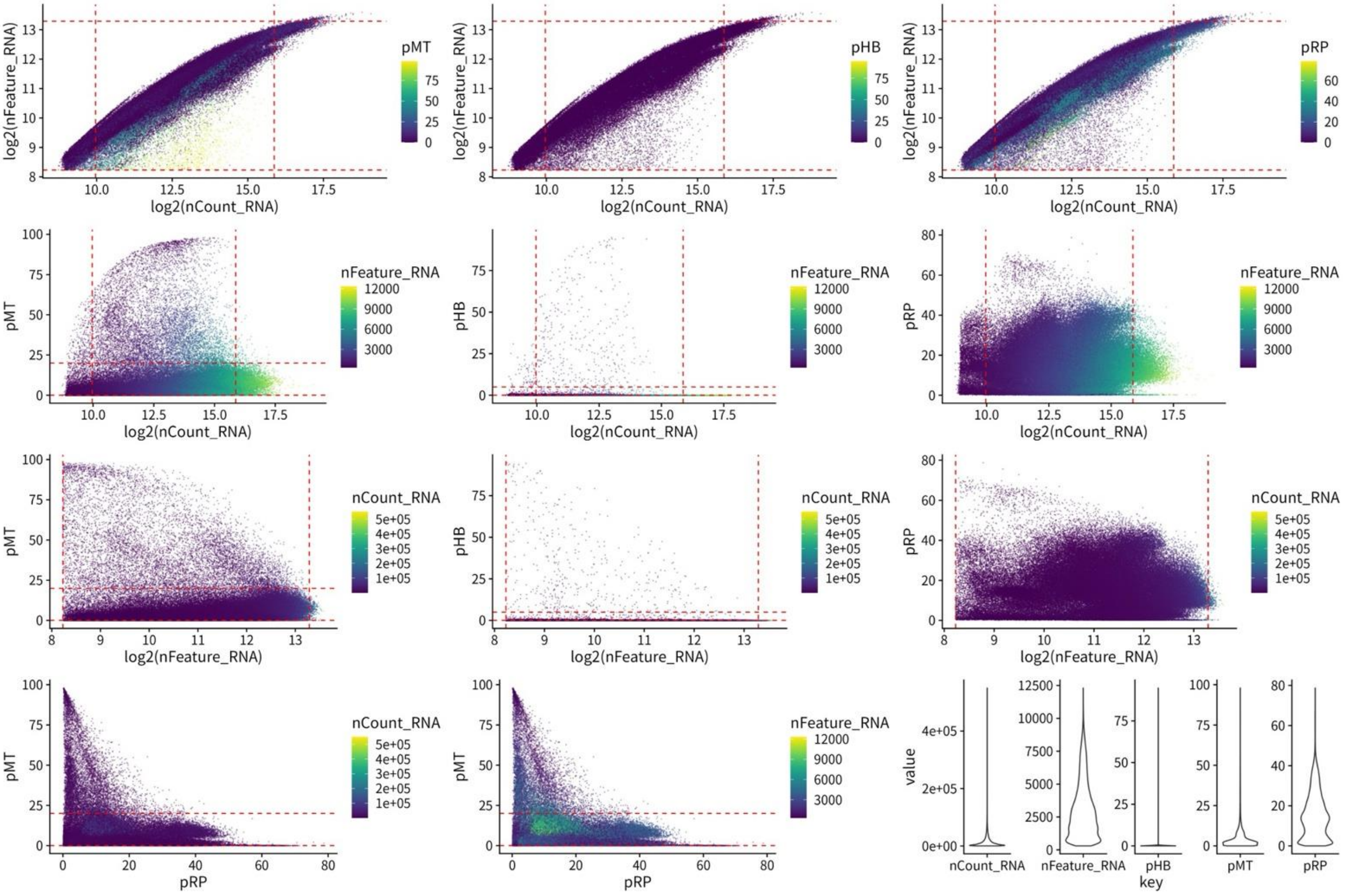


**Supplementary Fig. 1| Schematic diagram of quality control of scRNA-seq data.**
The dots represent each cell and the red dotted lines represent the thresholds for quality control.

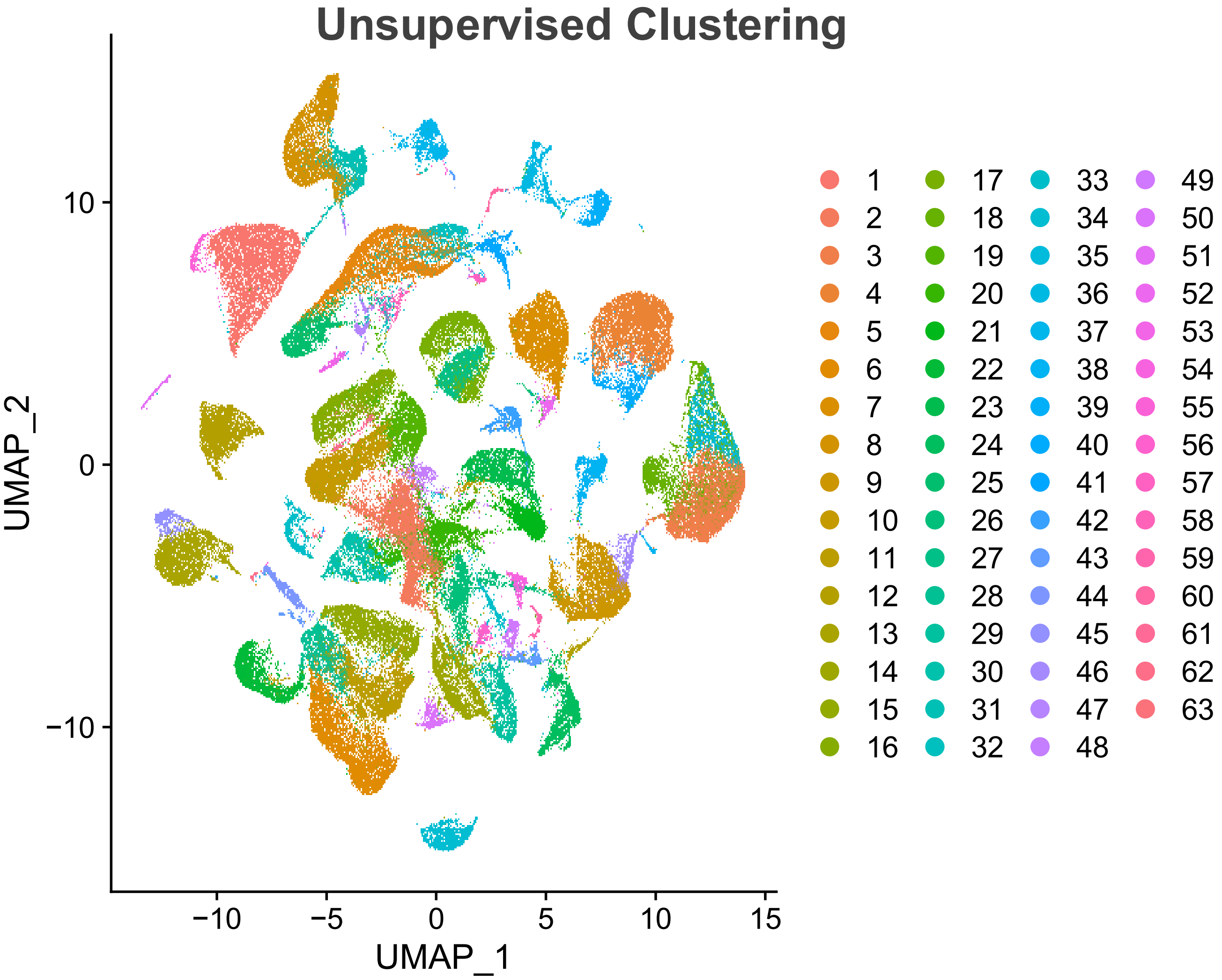


**Supplementary Fig. 2| UMAP for Unsupervised Clustering**
The dots represent each cell and the results of the unsupervised clustering are indicated by colour, a total of 63 clusters were obtained.

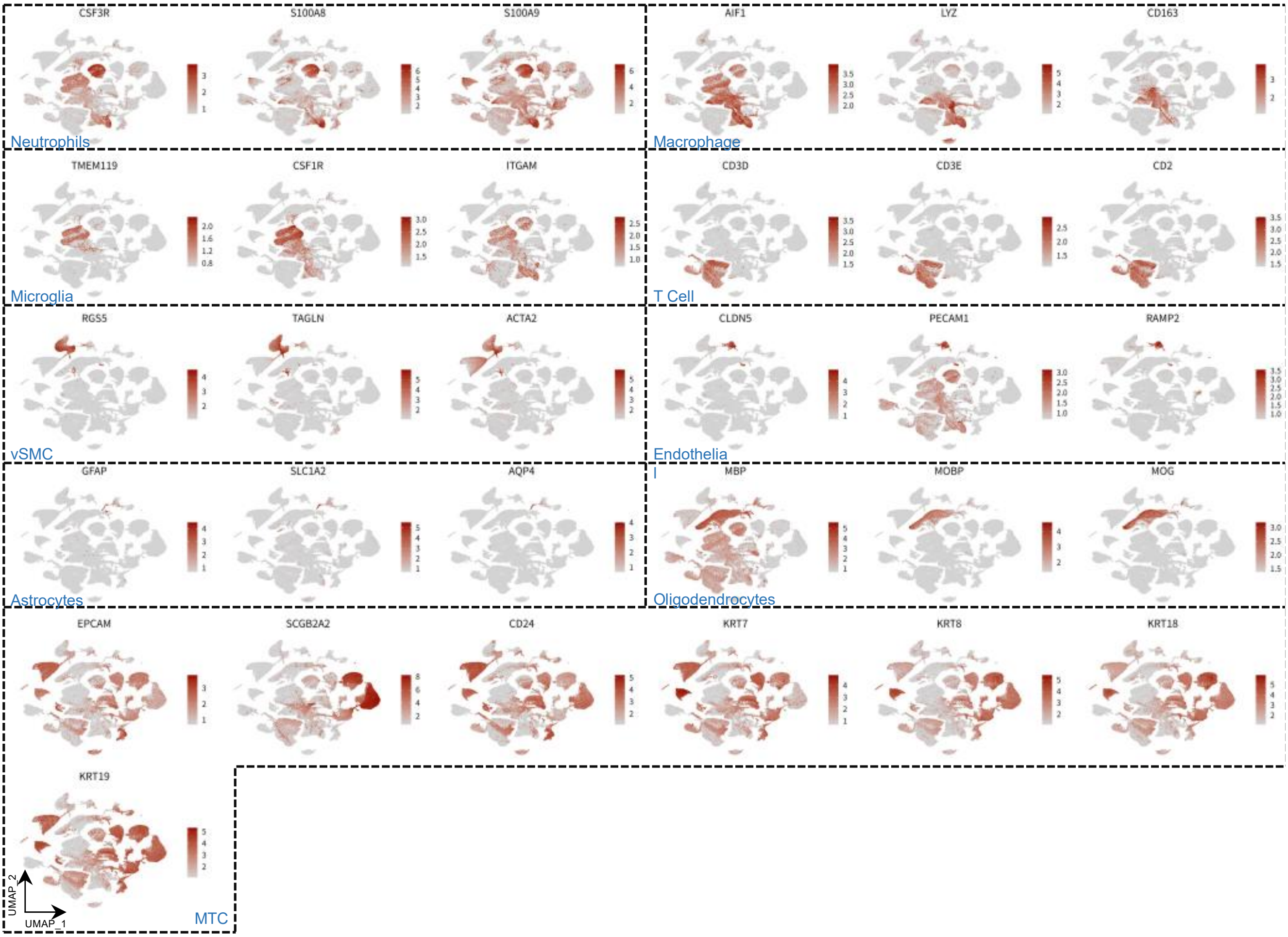


**Supplementary Fig. 3| Feature plots for each of marker gene**
Gene expression level for each of marker gene among all cell clusters.

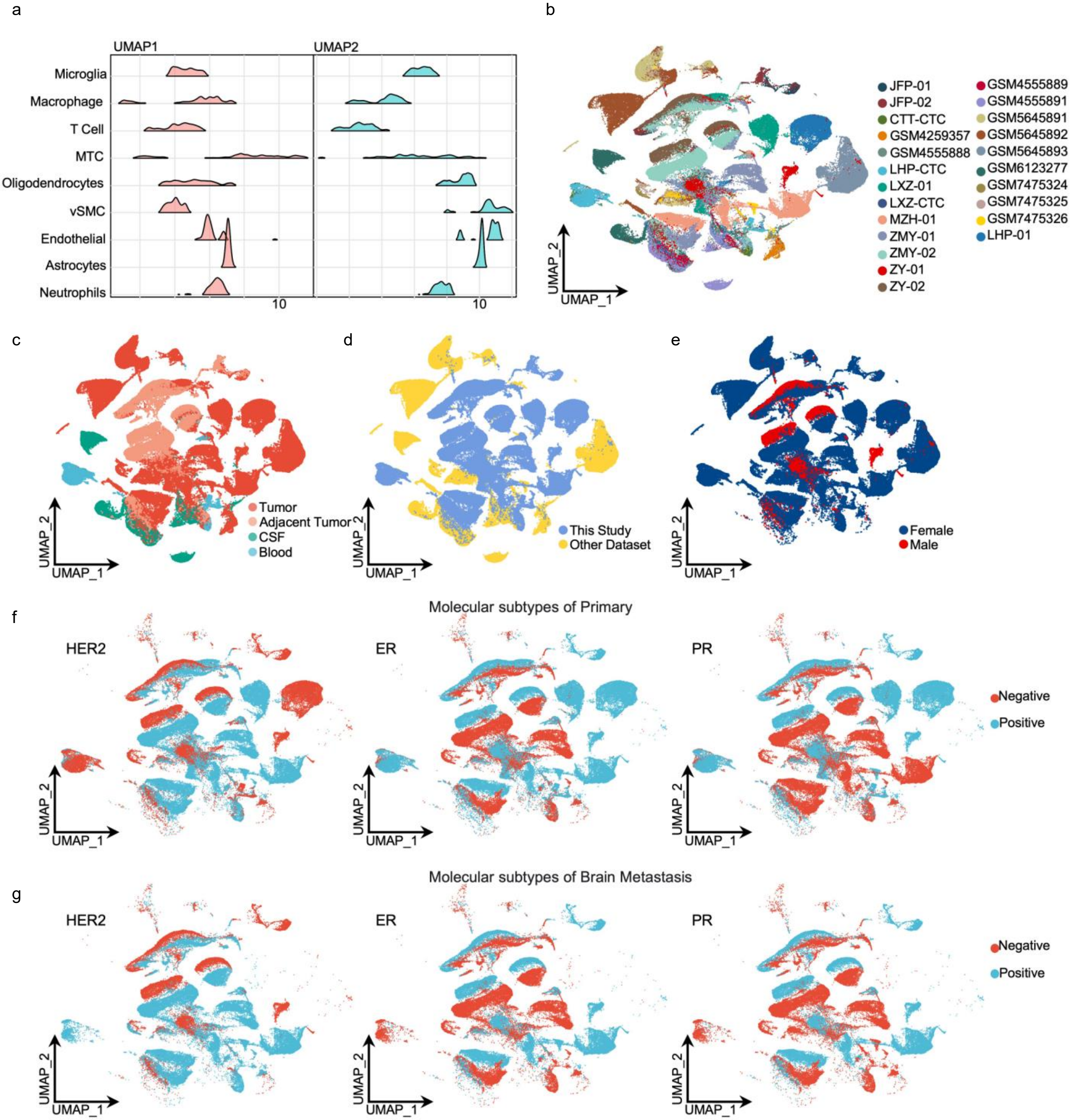


**Supplementary Fig. 4 | UMAP information for heterogeneity.** a, Coordinate density of all cell types in 2D UMAP. b, UMAP plots show the distribution of samples. Dots represent individual cells. c, UMAP plots show the distribution of sapling sites. Dots represent individual cells. d, UMAP plots show the distribution of dataset sources. Dots represent individual cells. e, UMAP plots show the distribution of gender. Dots represent individual cells. f, UMAP plots show the distribution of molecular subtypes of primary. Dots represent individual cells. g, UMAP plots show the distribution of molecular subtypes of brain metastasis. Dots represent individual cells.

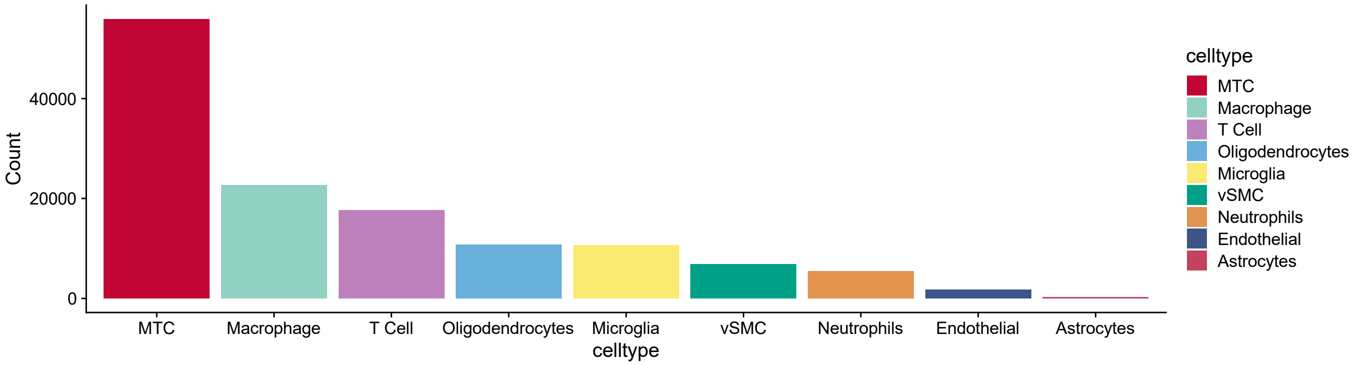


**Supplementary Fig. 5| Summary for major cell type in scRNA-seq**
Barplot for all types of cells. Colored by cell type.

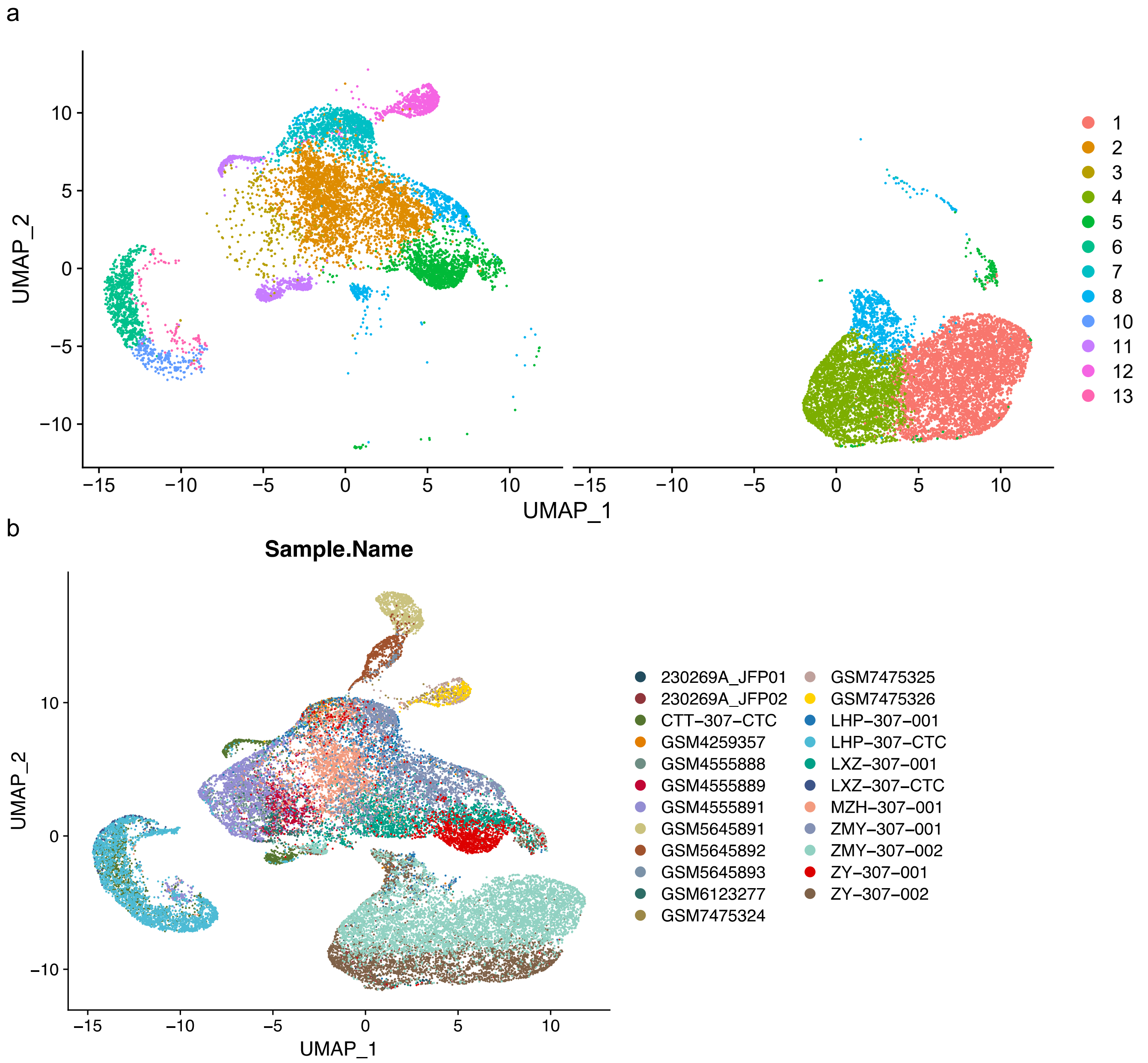


**Supplementary Fig. 6| UMAP plots for macrophages and microglia**
a, UMAP plots show the distribution of microglia (left) versus macrophages (right). b, UMAP plot shows the total distribution of microglia and macrophages, colored by sample

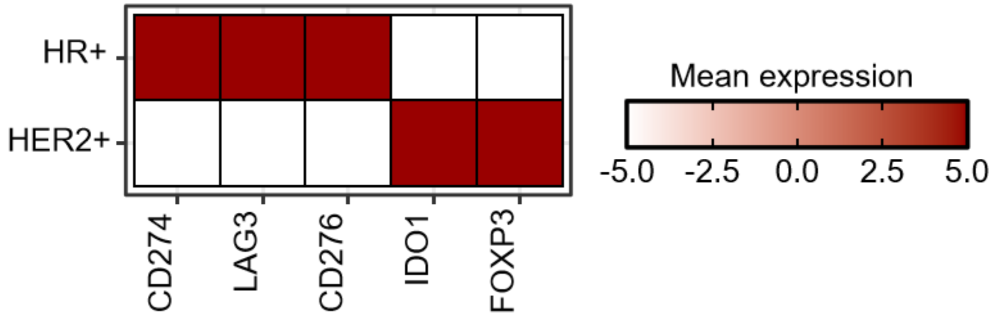


**Supplementary Fig. 7| TIME in brain metastasis of different molecular subtypes.**
Relative mean expression of immune checkpoint gene in different molecular subtypes of patients

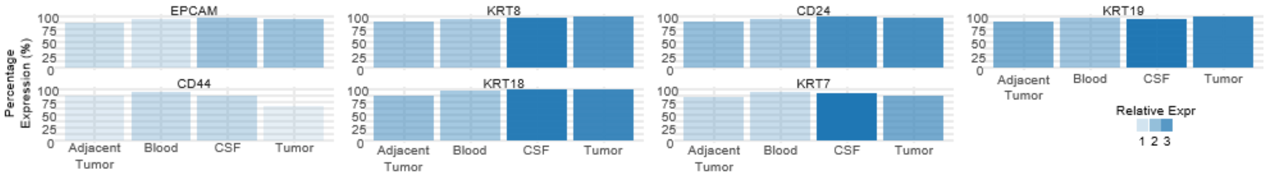


**Supplementary Fig. 8| Bio-markers for MTCs.**

Bar plots show the percentage expression in each MTCs subtypes, colored by relative mean expression.

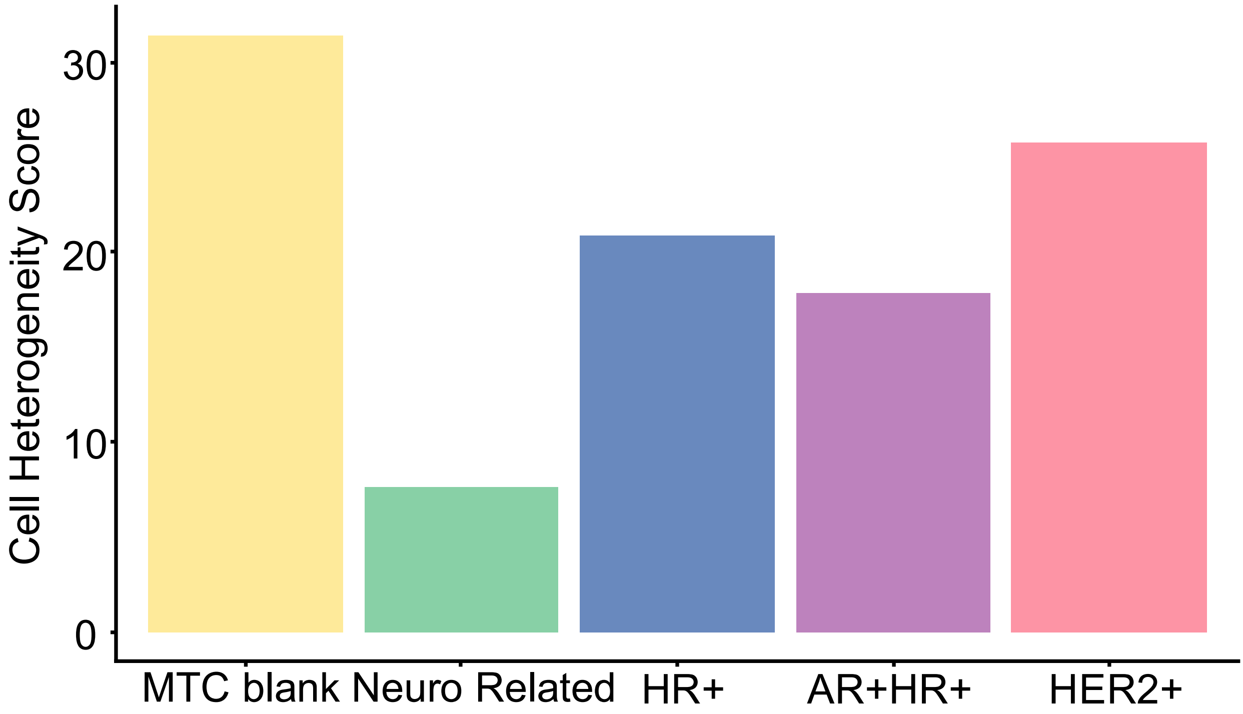


**Supplementary Fig. 9| Heterogeneity score**
Barplot for the cell heterogeneity score of all MTCs subtypes. Y-axis represent mean cell heterogeneity score.

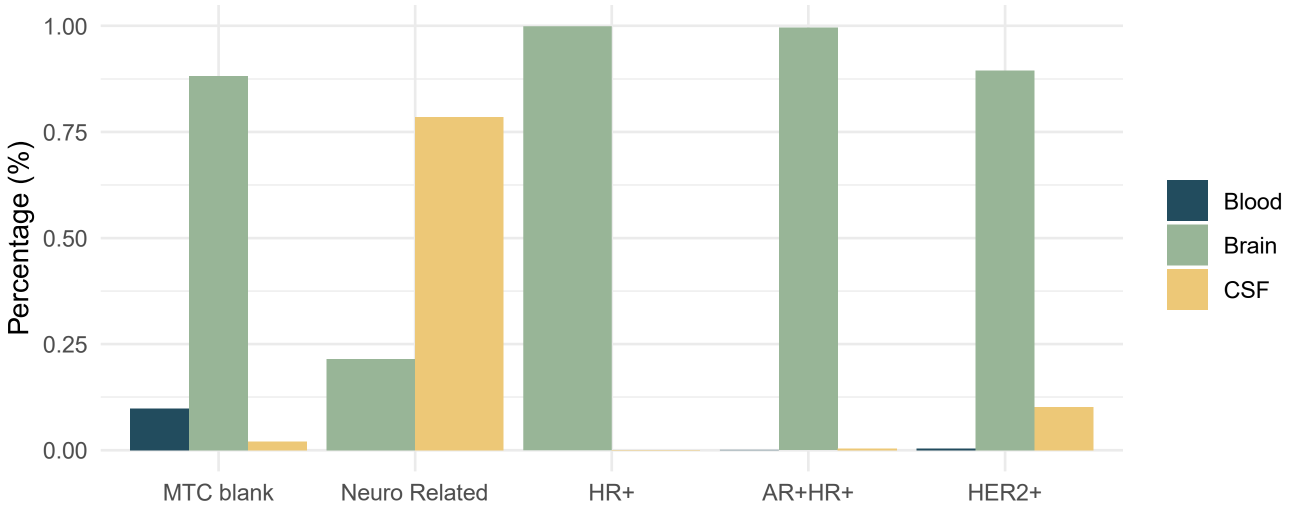


**Supplementary Fig. 10| The distribution of MTCs**
Barplot shows the distribution of all MTCs subtypes for different sampling sites.

a

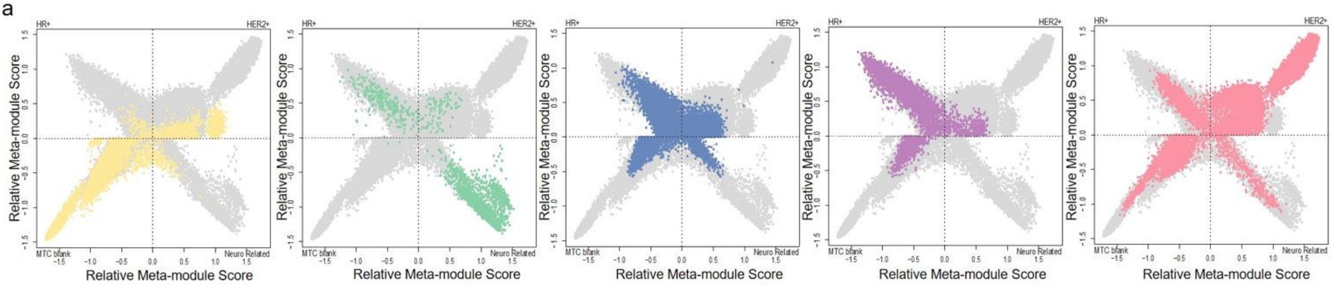


b

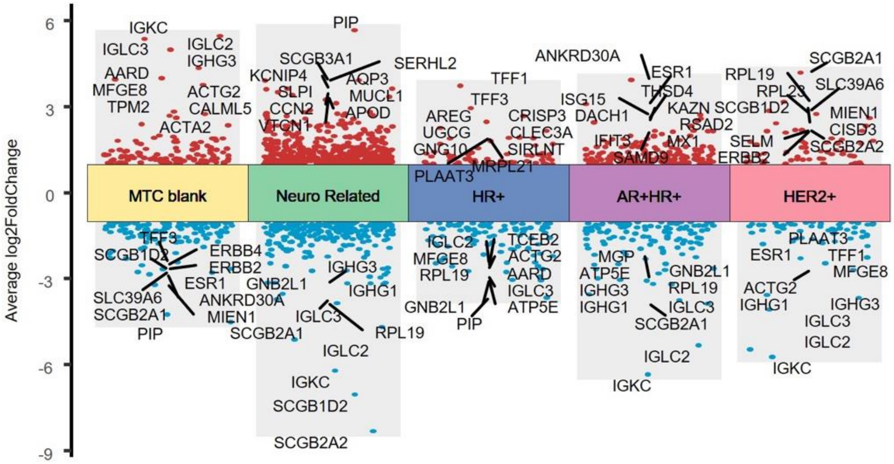


**Supplementary Fig. 11| Meta modules and Differential expression biological markers.**

a, Two-dimensional butterfly plot visualization of MTCs molecular subtype per meta-module score.

b, Volcano plot showing statistically significant differentially expressed markers in molecular subtypes of MTCs. The dots represent genes, with the red ones being relatively highly expressed genes and the blue ones representing relatively lowly expressed genes

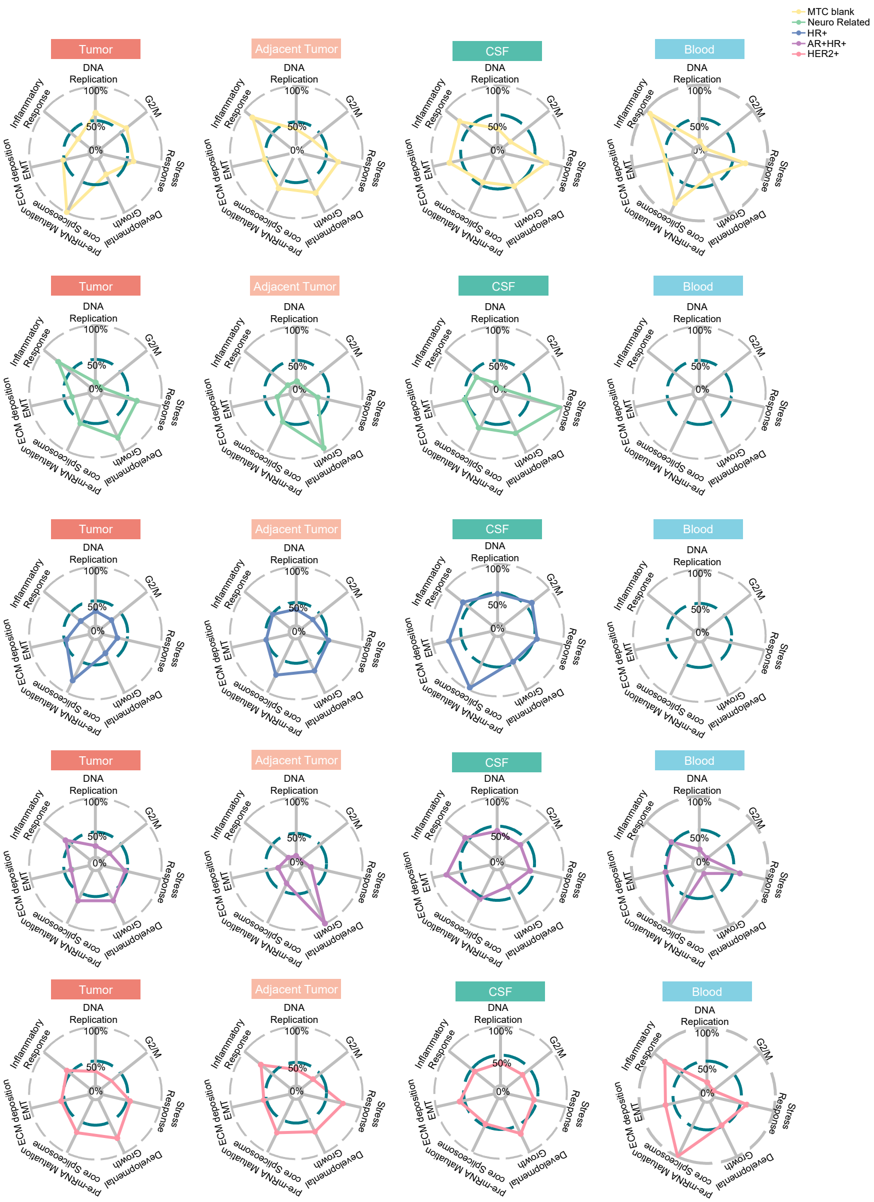


**Supplementary Fig. 12| Radar plots for cancer related pathways**

Radar plots show cancer related pathways enriched for five subtypes of MTCs in different sampling sites.

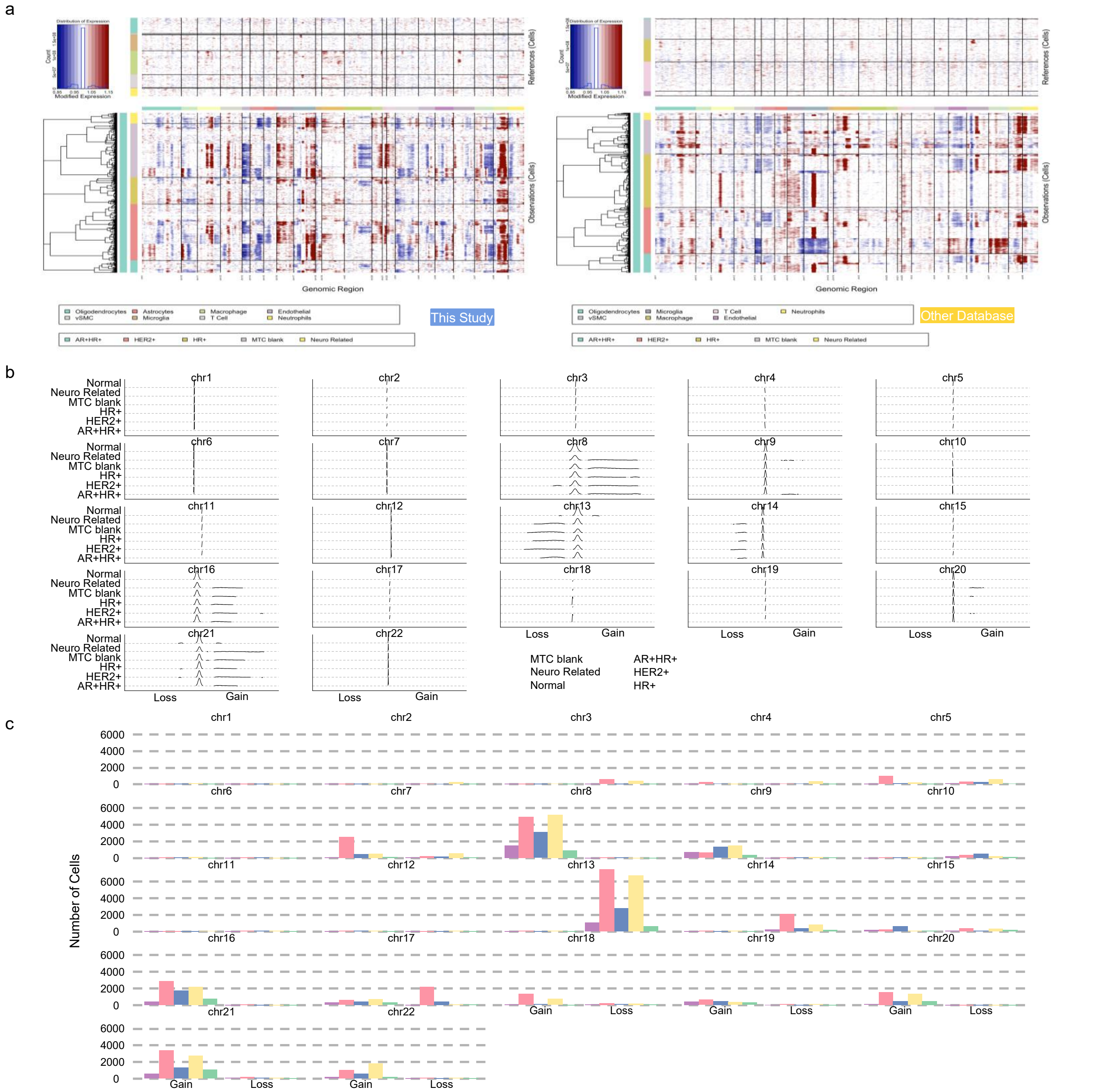


**Supplementary Fig. 13| Chromosome characteristic in breast cancer cells for brain metastasis. a**, CNV profiles inferred from scRNA-seq data for each cell. All no-MTCs as references, MTCs as observation. **b**, Ridgeline plots show the distribution of CNV scores of all chromosomes (calculated by inferCNV) in different subtypes of MTCs. **c**, Barplot shows the count of CNV cells in different chromosomes colored by MTCs subtypes.

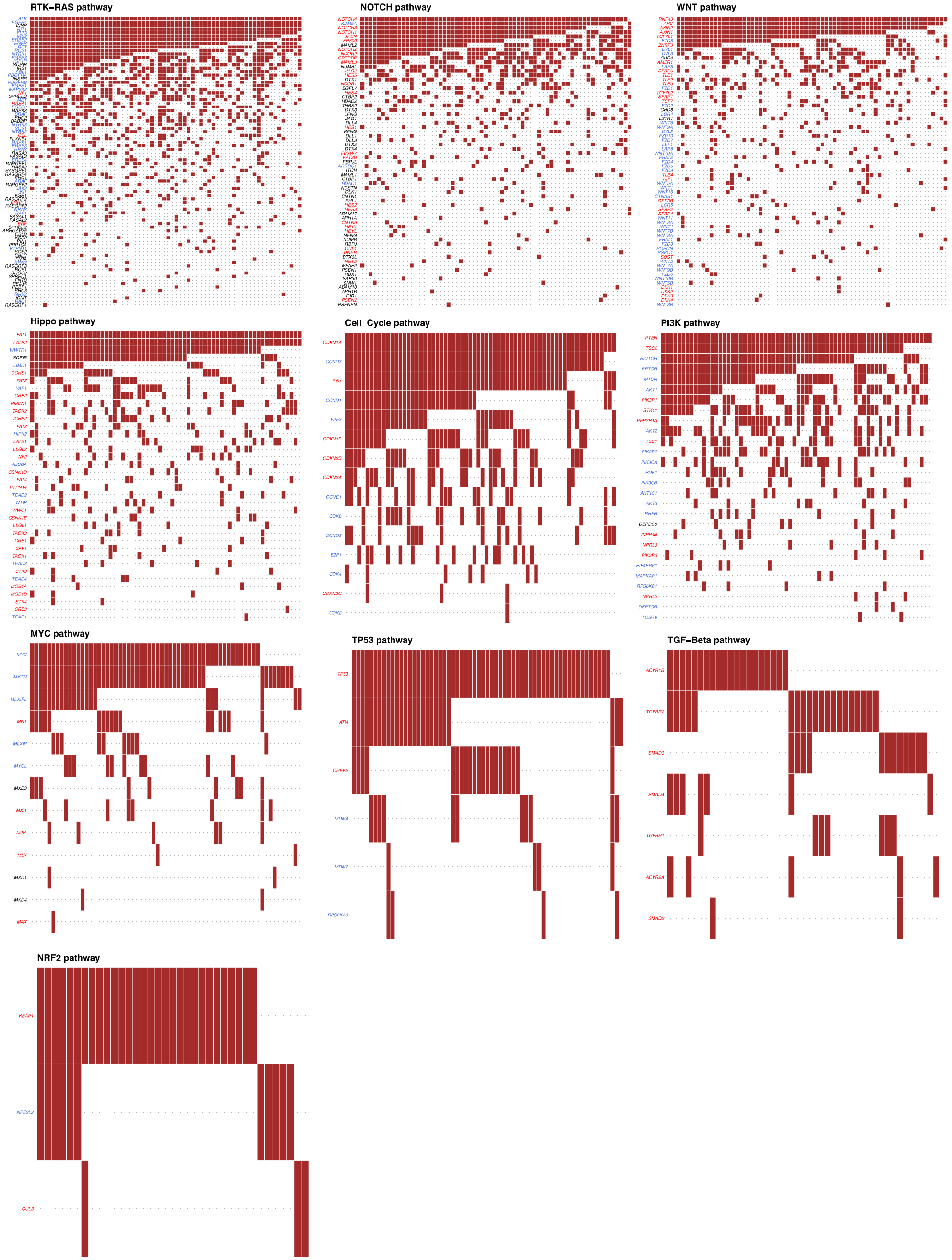


**Supplementary Fig. 14| Genetic mutations in the pathway**

Waterfall plots show the mutations of all genes in the pathway, where genes labelled in red are oncogenes and those labelled in blue are oncogenes.

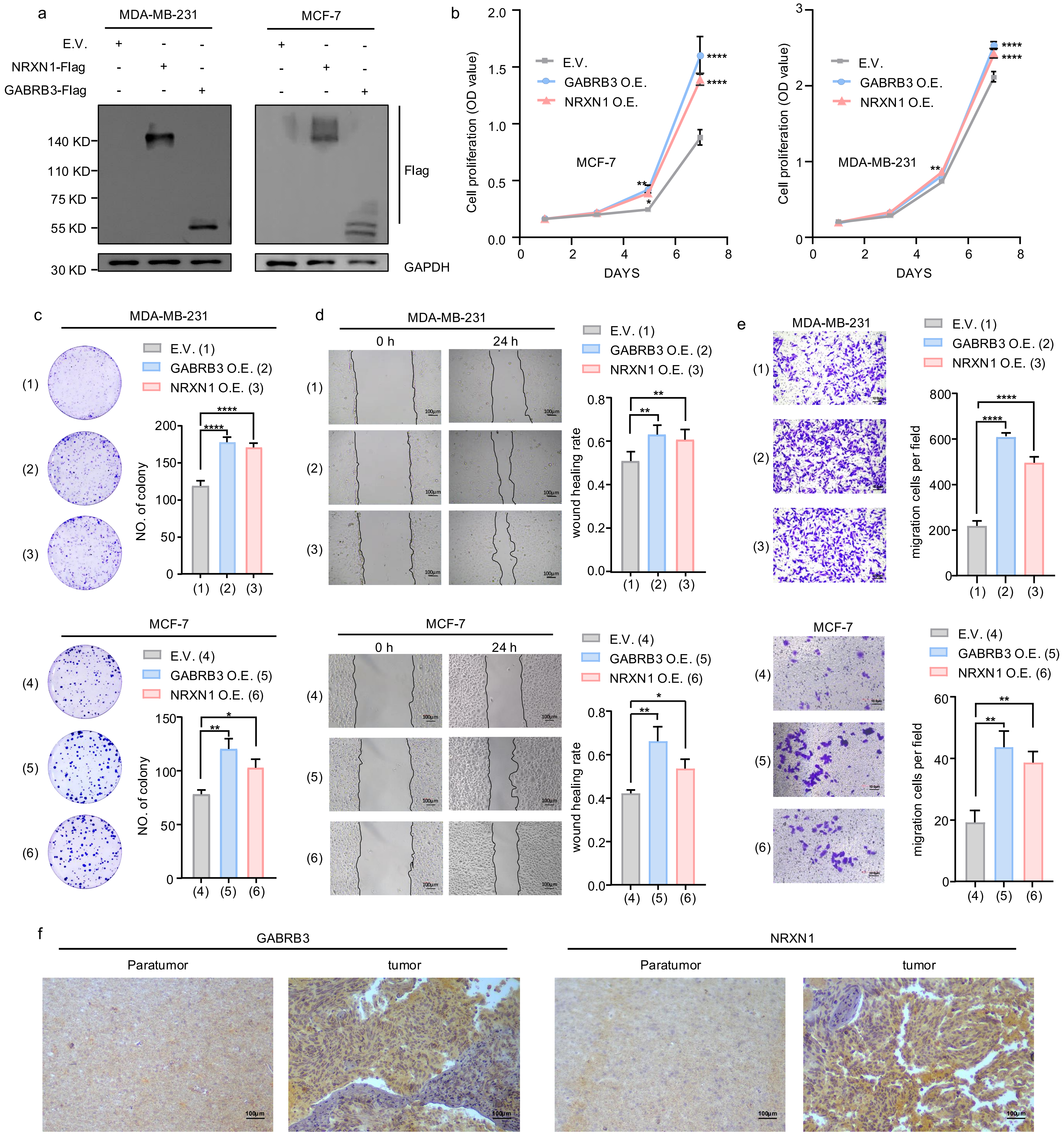


**Supplementary Fig. 15| NRXN1 and GABRB3 promotes the malignant behavior of HER2-negative breast cancer cells.** a, Human HER2- negative breast cancer cell lines MDA-MB-231 and MCF-7 were transfected with empty vector or NRXN1-flag and GABRB3-flag expression vector. WB were performed to confirm the protein levels. b, Cell growth ability was assessed using CCK8 assays with absorbance measured at 450 nm (OD450) with a microplate reader at the specified time intervals. c, Colony formation assay: Representative images (left panels) show colonies in the culture plates. The bar graph (right panels) quantifies the colonies. d, Wound healing assay: Representative images (left panels) show relative cell migration, with the bar graph (right panels) indicating the healing rate. e, Transwell invasion assay: Representative images (left panels) show relative cell invasion. The bar graph (right panel) quantifies the invasion. f, Representative IHC staining of GABRB3 and NRXN1 in in the tumor regions of brain metastatic tissues and their adjacent normal brain tissues. *P<0.05; **P<0.01; ****P<0.0001.
