# Supplementary figures and images for: Single-cell profiling reveals distinct populations of tumor-associated macrophages and metastatic tumor cells in breast cancer brain metastasis
Source: Cell Death Dis. 2026 Apr 25;17(1):553. doi: 10.1038/s41419-026-08807-w (PMC13247122; doi:10.1038/s41419-026-08807-w)

FLAG

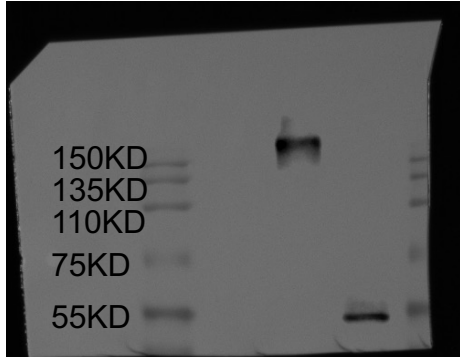

FLAG

150KD  
135KD  
110KD  
75KD  
55KD

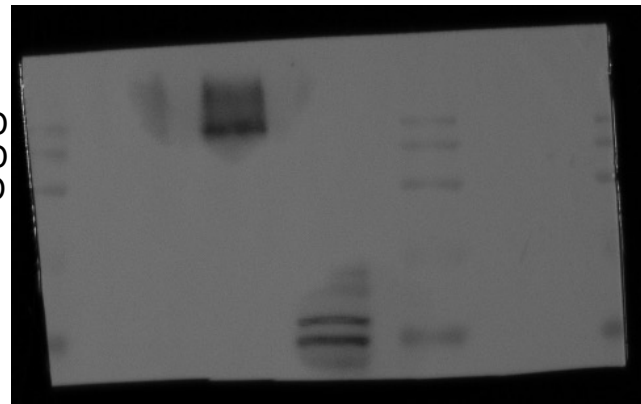

GAPDH

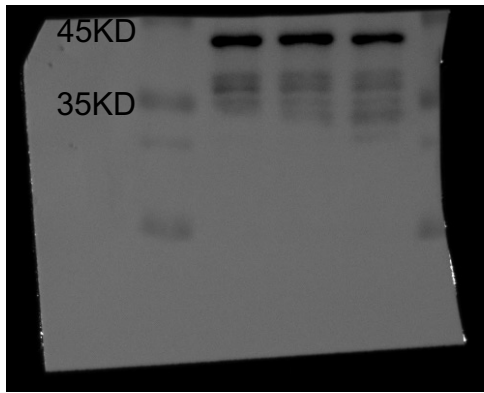

GAPDH

45KD  
35KD

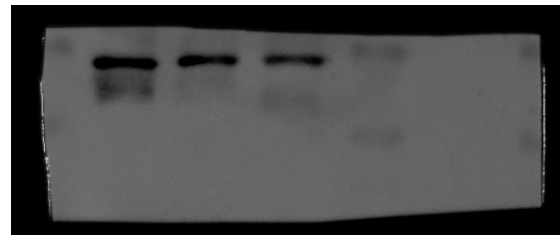

Supplement: Supplementary file 16 — Original Data [file 41419_2026_8807_MOESM16_ESM.pdf]
